# Exploring genetic association of insomnia with allergic disease and asthma: a bidirectional Mendelian randomization study

**DOI:** 10.1186/s12931-022-02009-6

**Published:** 2022-04-07

**Authors:** Rong Li, Yiting Chen, Anda Zhao, Lili Huang, Zichong Long, Wenhui Kang, Yong Yin, Shilu Tong, Yongmei Guo, Shenghui Li

**Affiliations:** 1 School of Public Health, Shanghai Jiao Tong University School of Medicine, 227 South Chongqing Road, Huangpu District, Shanghai, 200025 China; 2 Shanghai Ninth People’s Hospital, Shanghai Jiao Tong University School of Medicine, Shanghai, China; 3 Shanghai Children’s Medical Center, Shanghai Jiao Tong University School of Medicine, Shanghai, China; 4 School of Public Health, Institute of Environment and Population Health, Anhui Medical University, Hefei, China; 5 School of Public Health and Social Work, Queensland University of Technology, Brisbane, Australia; 6 Department of Neurology, Shuguang Hospital Affiliated to Shanghai University of Traditional Chinese Medicine, 528 Zhangheng Road, Pudong District, Shanghai, 201203 China; 7 MOE-Shanghai Key Laboratory of Children’s Environmental Health, Shanghai Jiao Tong University School of Medicine, Shanghai, China

**Keywords:** Mendelian randomization, Single nucleotide polymorphisms, Insomnia, Allergic disease, Asthma

## Abstract

**Background:**

Insomnia is highly prevalent among patients with allergic disease and asthma; however, few studies have investigated their causal relationship. We aim to explore the causal association between insomnia and allergic disease/asthma by performing bidirectional Mendelian randomization (MR) study.

**Methods:**

Instrumental variables were constructed using single nucleotide polymorphisms (SNPs). Summary statistics for insomnia, allergic disease, and asthma were obtained from four large-scale genome-wide association studies (GWAS) of European ancestry. The pleiotropy analysis was applied by using the MR-Egger intercept test and the MR pleiotropy residual sum and outlier (MR-PRESSO) test. MR analyses were conducted by using inverse variance weighted (IVW), weighted median, and MR-Egger method.

**Results:**

Based on the multiplicative random effects IVW method, the MR analysis showed that genetically predicted insomnia was causally associated with an increased risk of allergic disease [odds ratio (*OR*) = 1.054, 95% confidence interval (*CI*) = 1.031–1.078, *P* = 3.817 × 10^–06^], asthma (*OR* = 1.043, *95% CI* = 1.010–1.077, *P* = 9.811 × 10^–03^), moderate-severe asthma (*OR* = 1.168, *95% CI* = 1.069–1.277, *P* = 6.234 × 10^–04^), and adult-onset asthma (*OR* = 1.086, *95% CI* = 1.037–1.138, *P* = 4.922 × 10^–04^). In bidirectional analyses, we did not find evidence supporting the reverse causality relations.

**Conclusions:**

Our MR study suggested that genetically predicted insomnia was the risk factor for allergic disease and asthma. Improving sleep quality could be one of the cornerstones in the prevention of allergic disease and asthma.

**Supplementary Information:**

The online version contains supplementary material available at 10.1186/s12931-022-02009-6.

## Introduction

Insomnia is a widespread sleep disorder, with an annual incidence of approximately 35–50% in general population [1, 2]. It is characterized by difficulty in initiating or maintaining sleep, awakening in the morning, or feeling of non-resistant sleep [1]. Insomnia can cause significant daytime symptoms and negative health outcomes, including fatigue, daytime sleepiness, impairment in cognitive performance, and mood disturbances [1, 3]. The detrimental effects of insomnia cardiovascular and nervous function have been recognized by prospective epidemiology studies [4, 5]. However, available data on the role of insomnia in allergic disease such as asthma is limited.

In recent decades, the prevalence of allergic disease has been increasing, and allergic disease has become a momentous public health problem [6]. In 2016, the number of people suffering from asthma worldwide was close to 339 million [7]. Insomnia is highly prevalent among patients with allergic disease, and it is reported that 44% to 70% of asthma patients and 33% to 87% of atopic dermatitis people have symptoms of insomnia [8, 9]. And the converse is also true that poor sleep itself could cause or worsen allergic disease [8–12]. Two longitudinal observational studies have provided supportive evidence that insomnia carried an increased risk of newly-onset asthma [8, 11]. It seemed that there is a bidirectional causal relationship between insomnia and atopic dermatitis [9], and mutual causality seem to be plausible when explaining the association between insomnia and allergic disease. However, the majority of epidemiological data on insomnia and allergic disease was embedded in an observational design, though these studies attempted to increase their credibility by adjusting confounding factors, which is less likely to fully account for confounding and reverse causation bias. Therefore, the causal directionality between insomnia and allergic disease remains unclear, and it warrants further exploration.

Mendelian randomization (MR) overcomes the limitations of observation methods by using genetic variants (mainly single nucleotide polymorphisms, SNPs) as instrumental variables (IVs) to assess the potential causal impact of exposure on the results. Genetic variants are randomly assigned at conception, reflecting the randomization process in controlled trials and limiting the impact of confounding always. Genetic variants precede the onset of disease, eliminating adverse events to causality. Moreover, the selected IV is related to exposure, but neither related to any confounding factors in the exposure-result relationship, nor is it related to the result by means other than exposure [13]. Thus, MR is an ideal technique to explore the causal relationship between insomnia and allergic disease.

In this study, based on the summary statistics from large genome-wide association studies (GWAS) datasets, we performed a bidirectional MR study to determine the causal associations of genetically predicted insomnia with allergic disease, asthma, and its phenotypes.

## Methods

### Study design

To assess whether insomnia is associated with allergic disease, asthma, and three phenotypes of asthma (including moderate-severe asthma, adult-onset asthma, and childhood asthma) and to assess the direction of association, we performed a bidirectional MR study using the most up-to-date publicly available GWASs. The MR approach was based on 3 assumptions: (1) The genetic variants used as IVs are associated with exposure; (2) The genetic variants are not associated with any confounders; (3) There is no direct correlation between genetic variation and the outcome, or any way other than the exposure to correlate with the outcome [14].

### Genetic associations with insomnia

Based on a large-scale GWAS, which includes 1,331,010 individuals of European ancestry (944,477 individuals from 23andMe, and 386,533 individuals from UK Biobank), 248 SNP associated with insomnia at the genome-wide significance level (*P* < 5 × 10^–8^) were extracted, which is capable of explaining 2.6% of the variance in insomnia [2]. Insomnia was a self-reported condition collected through a electronic questionnaire integrated into UK Biobank’s touchscreen devices and an online questionnaire from 23andMe. The questionnaire of the UK Biobank has higher sensitivity (98%) and specificity (96%) when comparing with the insomnia severity index or the Pittsburgh sleep quality index. The phenotypes in the UK Biobank and 23andMe have acceptable sensitivity and specificity (> 80%) compared to those identified by structured interviews [15]. The association tests were adjusted for age, sex, genotype array, and 10 genetic principal components in the UK Biobank, and age, sex, the top 5 principal components in 23andMe.

### Genetic associations with allergic disease

Genetic association data for the allergic disease came from a publicly available GWAS (meta-analysis of results from the 13 studies) with the largest sample sizes hitherto [16], and they identified 136 independent risk variants (*P* < 3 × 10^−8^), including 73 not previously reported, which implicate 132 nearby genes in allergic disease pathophysiology. This study included 180,129 cases with self-reported suffering from asthma and/or hay fever and/or eczema, and 180,709 controls without suffering from any of these diseases/symptoms, all of European ancestry [16].

### Genetic associations with asthma, adult-onset asthma and childhood asthma

Summary-level statistics for asthma, adult-onset asthma, and childhood asthma were derived from the UK Biobank, a cohort study involving approximately 500,000 adults aged 37–73 years enrolled between 2006 and 2010 [17]. Asthma cases in the UK Biobank were identified by participant questionnaires whether they had ever been diagnosed with asthma by doctor. The current study limited analysis to 394,283 subjects of European ancestry to minimize population stratification (46,802 asthma patients, and 347,481 controls) [18]. Adult-onset asthma was defined as the age of onse 26 years (22,296 cases and 347,481 controls), childhood asthma was defined as the age of onset ≤ 12 years (9676 cases and 347,481 controls) [18].

### Genetic associations with moderate-severe asthma

We selected a large-scale GWAS dataset of 30,810 individuals who have European ancestry, including 5135 moderate-to-severe asthma cases and 25,675 controls [19]. These moderate-to-severe asthma cases came from the Genetics of Asthma Severity and Phenotype study (GASP, *n* = 1858), the Unbiased Biomarker Prediction of respiratory diseases outcomes project (U-BIOPRED, *n* = 281), and the UK Biobank (*n* = 2996). The selected controls were from U-BIOPRED (*n* = 75) and UK Biobank (*n* = 25,600). Patients in GASP and U-BIOPRED were evaluated using clinical records according to the 2014 guidelines of the British Thoracic Society. In UK Biobank, cases of moderate to severe asthma are based on a physician’s diagnosis.

The summary statistics excluded the 23andMe sample, intending to protect the privacy of the 23andMe research participants. Detailed information on data sources was shown in Additional file 1: Table S1.

### Selection of genetic instrument

Based on European ancestry, we used cluster functions implemented in the TwoSampleMR package to assess linkage disequilibrium between loci (LD), 207 genome-wide significance level (*P* < 5 × 10^–8^) and independent SNPs were proposed as IVs for insomnia, 98 SNPs for allergic disease, 86 SNPs for asthma, and 12 SNPs for moderate-severe asthma (r^2^ < 0.01 and clump window > 10,000 kb) (Additional file 2: Table S2). Bidirectional analysis for the adult-onset asthma and childhood asthma could not be conducted due to insufficient independent SNPs as IVs (0 and 1 SNP, respectively). To evaluate the strength of IV, we use the following formula to calculate the F statistic for each SNP. F statistic = R^2^ × (N − 2)/(1 − R^2^), where R^2^ is the phenotypic variance explained by each genetic variation in the exposure, and N is the sample size [20]. IVs with F-statistic < 10 were considered weak instruments [21]. Subsequently, these SNPs were harmonized in summary data of outcomes GWAS in the TwoSampleMR package (Additional file 2: Table S3).

### Statistical analysis

To evaluate the causal directionality between insomnia and allergic disease, asthma and it’s three phenotypes, we performed a bidirectional MR study using three models, including: (1) the inverse variance weighted (IVW) model; (2) the weighted median model; and (3) the MR-Egger regression model. The IVW model took a meta-analysis approach to combine Wald estimates for each SNP to get the overall estimates of the effect. If there is no horizontal pleiotropy and heterogeneity, an unbiased causal estimate could be obtained by IVW linear regression, and fixed and multiplicative random effects IVW approaches are available [22]. Due to the heterogeneity of causal estimates of different variants, the multiplicative random effects model would be more appropriate than the fixed effects model, so we used the former as the main analysis method [23]. The weighted median model can provide a consistent estimate of causality when at least 50% of genetic IVs are effective [24]. The advantage of the MR-Egger regression model is that it evaluates the null causal hypothesis under the assumption of Instrument Strength Independent of Direct Effect. Even if all SNPs included in the selection are invalid, MR-Egger can still provide a robust unbiased estimate [25, 26].

An important prerequisite for the MR approach is that exposure-related SNPs affect allergic disease, asthma, and its phenotypes, only through insomnia itself [25]. To assess whether IVs affect the level of pleiotropic effects of outcomes through more than one biological pathway, we used MR-Egger regression and MR-PRESSO (Mendelian Randomization Pleiotropy RESidual Sum and Outlier) to test for evidence of pleiotropy [25]. MR-Egger regression intercept that deviates from the origin may provide evidence for potential multiple pleiotropy effects in genetic IVs [26]. MR-PRESSO is a method that allows for the evaluation of horizontal pleiotropy in multi-instrument Mendelian Randomization utilizing genome-wide summary association statistics, and the MR-PRESSO global test is used to detect the horizontal pleiotropy [27]. The IVW and MR-Egger regression were used to detect potential heterogeneity among causal effects of different variants, the heterogeneities were quantified by the Cochran Q statistic, and I^2^ statistic [28]. The leave-one-out sensitivity method was performed to compute whether random estimates were affected by an individual genetic locus. For further interpretation, scatterplots, forest plots, and funnel plots were also produced.

Bonferroni method was performed to correct for multiple testing. The association with two-sided *P*-values < 0.01 (0.05/5, insomnia as outcome) and *P*-values < 0.01 (0.05/3, allergic disease, asthma, moderated-severe asthma as outcome) were deemed statistically significant, *P*-values between 0.05 and 0.01 were regarded as suggestive evidence of association. Besides, other statistical tests were two-sided and the statistical significance was set at the level of *P* < 0.05. MR analysis was performed using TwoSampleMR packages (version 0.5.6) in R (version 4.1.1).

## Results

### Genetically predicted insomnia on the risk of allergic disease, asthma and its phenotypes

**Fig. 1:**
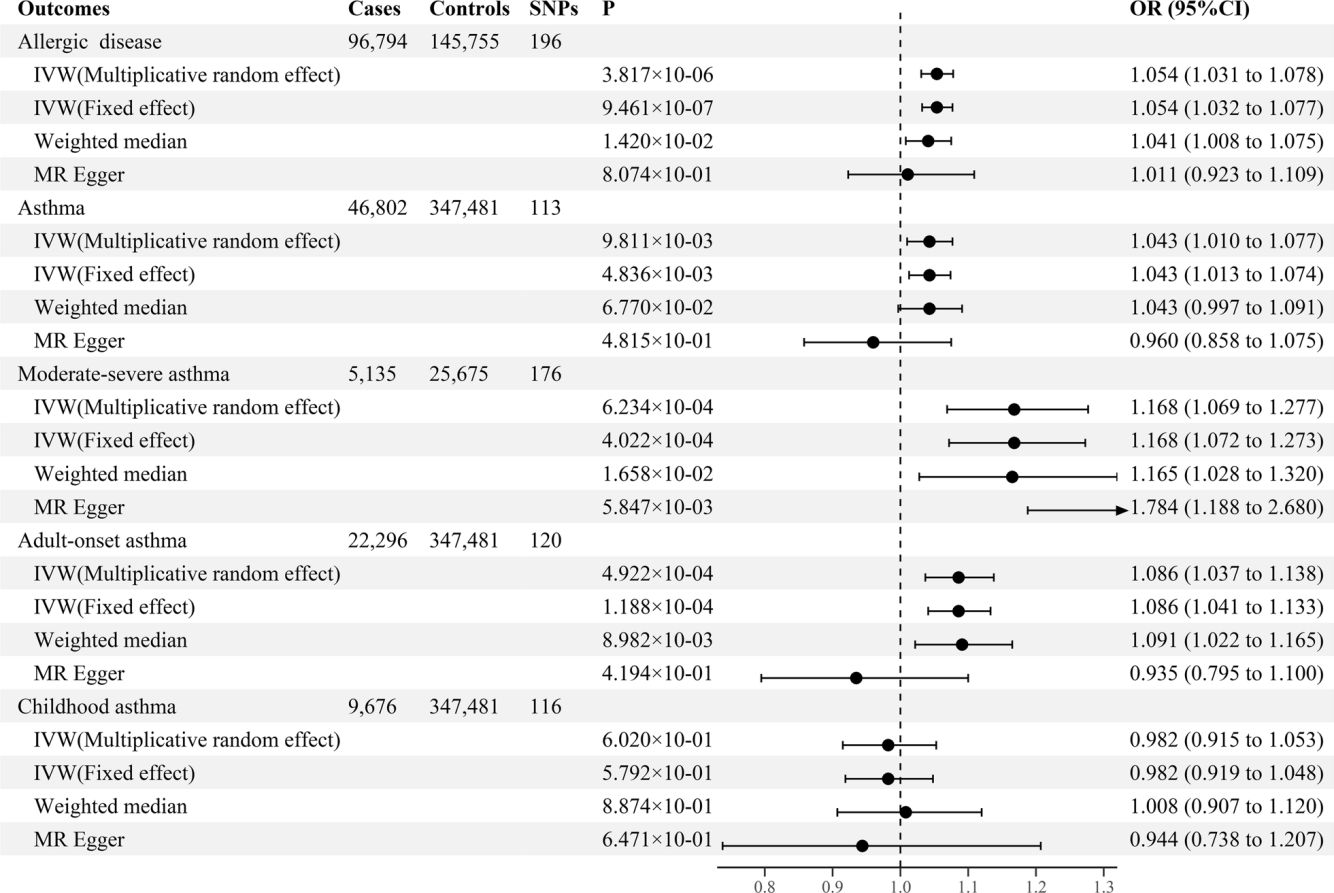
Odds ratios for the associations between genetically predicted insomnia and risk of allergic disease, asthma and its phenotypes. SNPs: the number of SNPs used as instrumental variables; P: P-value of the causal estimate; OR: odds ratio; CI: confidence interval

**Fig. 2:**
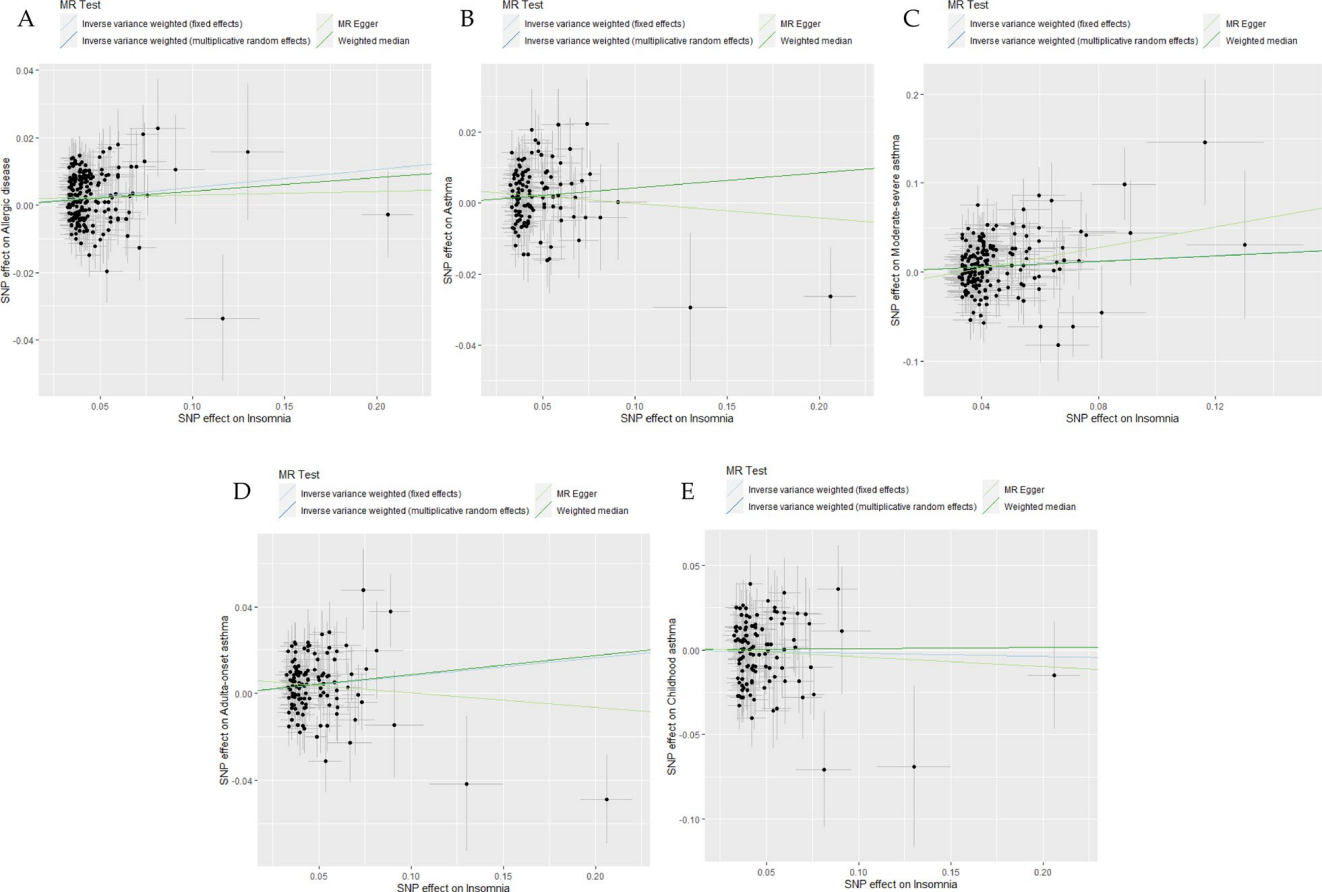
Scatterplots of potential effects of SNPs on insomnia versus allergic disease, asthma, and its phenotypes. **A** Allergic disease; **B** asthma; **C** moderate–severe asthma; **D** adult-onset asthma; **E** childhood asthma. Analyses were conducted using the fixed and multiplicative random effects IVW, MR-Egger, Weighted median methods. The slope of each line corresponding to the estimated MR effect per method

Based on the multiplicative random effects IVW method, the MR analysis showed that genetically predicted insomnia increased the risk of allergic disease (odds ratio [*OR*] = 1.054; *95%* confidence interval [*CI*] = 1.031–1.078, *P* = 3.817 × 10^–06^), and the similar results were also obtained in the weighted median model (Fig. 1, and Additional file 1: Table S4). Moreover, we observed causal effects of genetically predicted insomnia on the risk of asthma, with odds ratios of 1.043 (*95% CI* 1.010–1.077, *P* = 9.811 × 10^–03^). As for asthma phenotypes, there were evidences to support causal associations between genetically predicted insomnia and both moderate-severe asthma (*OR* = 1.168, *95% CI* = 1.069–1.277, *P* = 6.234 × 10^–04^) and adult-onset asthma (*OR* = 1.086, *95% CI* = 1.037–1.138, *P* = 4.922 × 10^–04^), which was similarly observed in the weighted median model, but MR-Egger regression estimate was only statistically significant in moderate-severe asthma. By contrast, there was no significant causal relationship between genetically predicted insomnia and childhood asthma (*OR* = 0.982, *95% CI* = 0.915–1.053, *P* = 0.602). The estimated effect sizes for SNPs of insomnia on allergic disease, asthma, and its phenotypes were displayed in scatter plot (Fig. 2).

### Genetically predicted allergic disease, asthma and moderate-severe asthma on the risk of insomnia

**Fig. 3:**
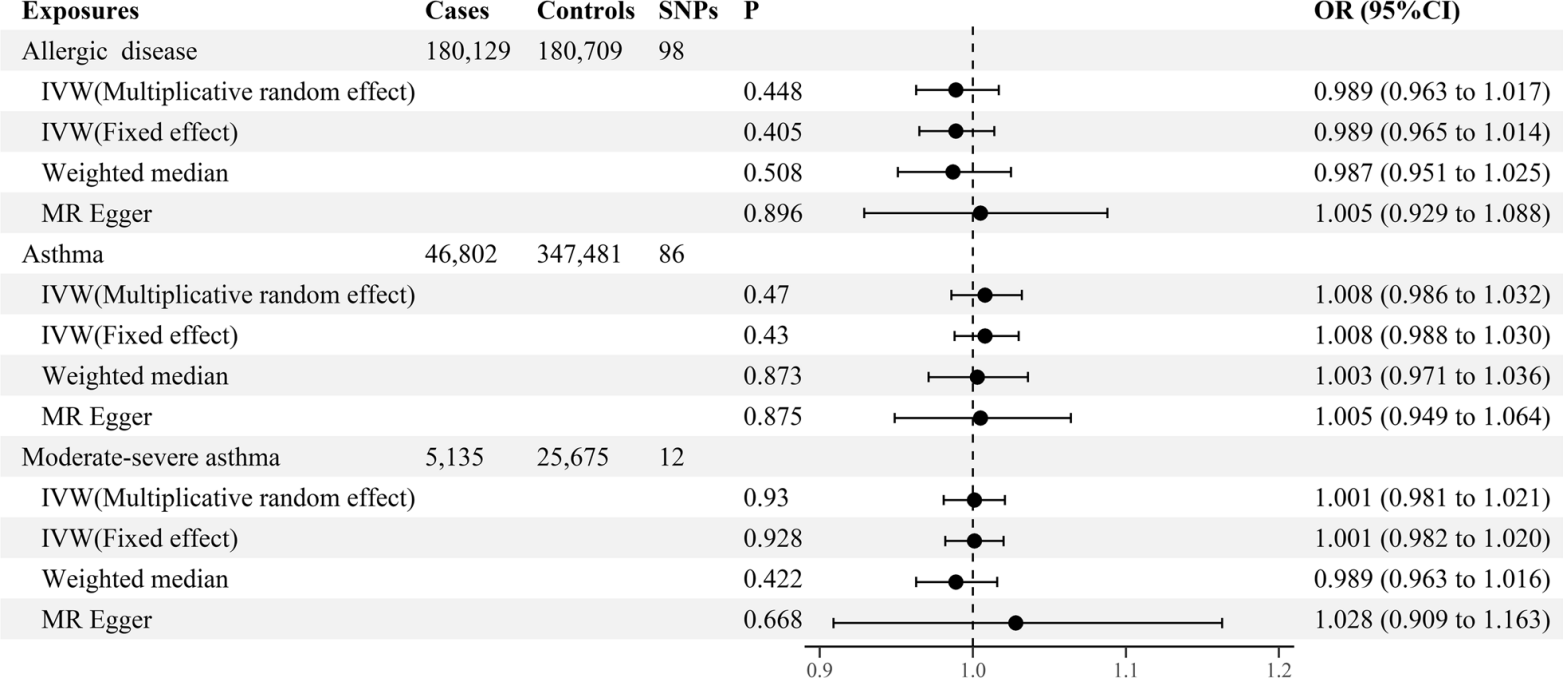
Odds ratios for the associations between genetically predicted allergic disease, asthma, moderate-severe asthma and risk of insomnia. SNPs: the number of SNPs used as instrumental variables; P: P-value of the causal estimate; OR: odds ratio; CI: confidence interval

**Fig. 4:**
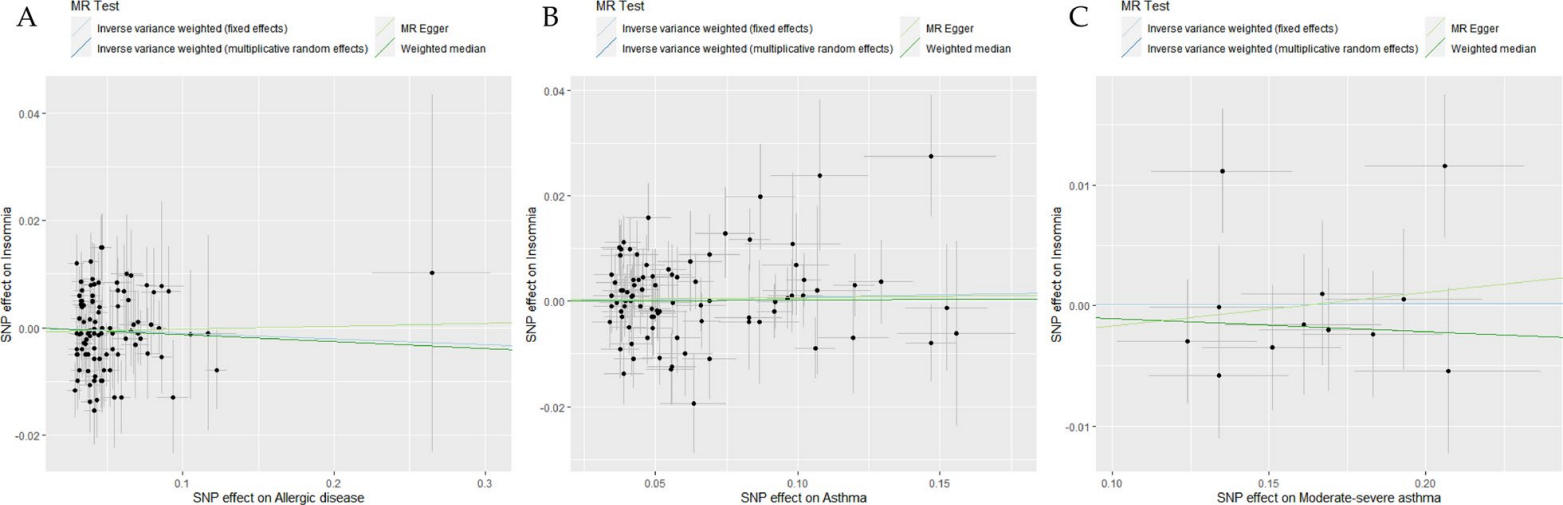
Scatterplots of potential effects of SNPs on allergic disease, asthma, moderate-severe asthma versus insomnia. **A** Allergic disease; **B** asthma; **C **moderate–severe asthma. Analyses were conducted using the fixed and multiplicative random effects IVW, MR-Egger, Weighted median methods. The slope of each line corresponding to the estimated MR effect per method

In bidirectional analyses, based on the multiplicative random effects IVW method, there was no evidence for potential causal effects of genetically predicted allergic disease, asthma and moderate-severe asthma on insomnia, with odds ratios of 0.989 (*95% CI* 0.963–1.017, *P* = 0.448), 1.008 (*95% CI* 0.986–1.032, *P* = 0.470), 1.001 (*95% CI* 0.981–1.021, *P* = 0.930), respectively (Fig. 3, and Additional file 1: Table S5). Similar results were obtained for the weighted median model and MR-Egger regression estimate. The estimated effect sizes for SNPs of allergic disease, asthma and moderate-severe asthma on insomnia were displayed in scatter plot (Fig. 4).

### Results for sensitivity analyses

Using the MR-Egger regression intercept test and MR-PRESSO global test, there was no evidence of horizontal pleiotropy for SNPs of insomnia as exposure with allergic disease, asthma and its phenotypes as outcomes (all *P* > 0.05) (Table 1). Cochran Q statistic and I^2^ statistic indicated low heterogeneity and more reliability of these SNPs (all *P* > 0.05). The funnel plots showed general symmetry, suggesting little evidence of heterogeneity (Additional file 1: Fig. S1). We performed the leave-one-out sensitivity analysis using conventional IVW methods, and the results were similar after removing single SNPs in the leave-one-out analysis, suggesting that no single SNP has an exorbitant influence on the overall estimates (Additional file 1: Figs. S2–S6). The forest plots were shown in Additional file 1: Figs. S7–S11.

**Table 1:** Pleiotropy and heterogeneity test of insomnia on allergic disease, asthma and its phenotypes

Outcomes	MR-Egger Test			Cochrane Q Test								MR-PRESSO Global Test	
				MR-Egger				IVW					
	Intercept	SE	*P*-value	Q	Q_df	*I*^2^	*P*-value	Q	Q_df	*I*^2^	*P*-value	RSSobs	*P*-value
Allergic disease	0.002	0.002	0.360	218.5	194	0.112	0.110	219.0	195	0.110	0.110	221.750	0.104
Asthma	0.004	0.003	0.135	130.7	111	0.151	0.098	133.3	112	0.160	0.083	136.229	0.071
Moderate-severe asthma	− 0.018	0.009	0.038	182.6	174	0.047	0.312	187.2	175	0.065	0.250	189.379	0.250
Adult-onset asthma	0.007	0.004	0.062	140.9	118	0.163	0.074	145.1	119	0.180	0.052	148.374	0.053
Childhood asthma	0.002	0.006	0.747	129.9	114	0.123	0.146	130.0	115	0.116	0.160	132.260	0.161

*SE* standard error, *df* degree of freedom

In bidirectional analyses, the MR-Egger regression intercept and MR-PRESSO global test also suggested that no evidence of horizontal pleiotropy for SNPs of allergic disease, asthma and moderate-severe asthma as exposures with insomnia as outcome (all *P* > 0.05) (Table 2). Likewise, no heterogeneity was found in the Cochran Q statistic, I^2^ statistic, and funnel plots (all *P* > 0.05). (Table 2, and Additional file 1: Figure S12) The leave-one-out analysis also suggested that no single SNP has an exorbitant influence on the overall estimates (Additional file 1: Figs. S13–S15). The forest plots were shown in Additional file 1: Fig. S16–S18.

**Table 2:** Pleiotropy and heterogeneity test of allergic disease, asthma and its phenotype on insomnia

Exposures	MR-Egger test			Cochrane Q test								MR-PRESSO global test	
				MR-Egger				IVW					
	Intercept	SE	*P*-value	Q	Q_df	*I*^2^	*P*-value	Q	Q_df	*I*^2^	*P*-value	RSSobs	*P*-value
Allergic disease	− 0.001	0.002	0.675	116.3	96	0.175	0.077	116.6	97	0.168	0.086	118.622	0.101
Asthma	0.0002	0.002	0.887	101.4	84	0.172	0.095	101.4	85	0.162	0.108	103.520	0.118
Moderate-severe asthma	− 0.004	0.010	0.674	11.4	10	0.140	0.325	11.6	11	0.055	0.391	13.901	0.380

*SE* standard error, *df* degree of freedom

## Discussion

To the best of our knowledge, this is the first bidirectional MR analysis to explore the potential causal relationship of insomnia with allergic disease, asthma, and its phenotypes. Our MR analyses demonstrated that genetically predicted insomnia was causally associated with increased risks of allergic disease, asthma and its phenotypes, moderate-severe asthma and adult-onset asthma but not childhood asthma. By contrast, the bidirectional analyses did not find evidence supporting genetically predicted allergic disease or asthma was causally associated with the risk of insomnia. Our findings, based on model of population genetic variation, pointed out the prophetic function of sleep disorder in individual’s susceptibility to allergic disease and asthma, but the opposition is not necessarily the case.

Although the previous observational studies were limited, the majority of them identified that insomnia was associated with the increased risks of allergic disease and asthma in both Western and Eastern populations, which was supported by the present MR study. A 5-year prospective cohort of 2316 middle-aged adults reported that patients with insomnia at baseline had a higher incidence of asthma and allergic rhinitis than those without insomnia [29]. Two cohort data from China and Norway also revealed insomnia was a risk factor for new-onset asthma [8, 11]. In addition, several studies have focused on other sleep traits, and the findings consistently demonstrated that poor sleep behavior has an impact on the development of allergic disease [30–34]. A study carried out in Britain explored the potential explanation why night shift work was associated with an increased risk of asthma, and it was proposed that circadian misalignment should be the main cause [30]. In terms of sleep duration, a clinical trial indicated that sleep restriction in asthmatics resulted in lower morning peak flow and forced expiratory volume in one second (FEV_1_) measurements [31]. Previous cross-sectional study has indicated that sleep duration < 7.8 h per night was associated with the higher odds of food and aeroallergens sensitization in rural Chinese adolescents [32]. In this study, we did not observe significant causal association between insomnia and childhood asthma. However, one retrospective cohort study demonstrated that frequent nocturnal awakening in early life is associated with subsequent asthma [34]. Given insomnia was not the pronounced sleep problem among children [35], evidence is quite limited to get clear knowledge; more studies are needed to focus on children population.

In individuals with allergic disease, insomnia usually appear to be frequent [8, 9]. Unfortunately, we could not find evidence that genetically predicted allergic disease, asthma, or moderate-to-severe asthma was causally linked with the increased risk of insomnia. The similar finding was observed by another MR analysis [12]. It seems that not all epidemiological link can be confirmed by MR study. A case in point is that most epidemiological data believed that there is a bidirectional causal relationship between insomnia and migraine, only a one-way link has been established by MR study, namely that genetically predicted insomnia is the risk for migraine [36]. In future studies, the causal associations between allergic disease, asthma, and insomnia need to be further explored by MR studies with larger GWAS samples.

Although the detailed pathophysiological bases remain to be resolved, several hypotheses have been set up to explain the link between insomnia and allergic disease. Insomnia or sleep loss could lead to a dysregulation of the immune system, which in turn reduces the number and the activity of natural killer cells and T cells in the body, which could increase individual’s susceptibility to allergens [37–39]. Insomnia is involved in the regulation of hypothalamus–pituitary–adrenal axis and the sympathetic nervous system, both of which together with tilting the basal gene expression profile toward increased pro-inflammatory [40, 41], activating β-adrenergic signaling, and inducing the increases in NF-κB, inflammatory gene expression, proinflammatory cytokines production, and systemic inflammation markers [41]. Meanwhile, insomnia could disturb the functional rhythm of regulatory T cells [39, 42], shifting the T helper 1 cell (TH1)/ TH2 balance toward TH2 dominance [43], and TH2 response contributes to allergic inflammatory disorders, including asthma, allergic rhinitis, atopic dermatitis, and anaphylaxis [44, 45]. Melatonin, a circadian regulating endocrine hormone, plays function in body’s immunomodulatory, antioxidant, and cytoprotective [46]. Insomnia suppresses melatonin production, thereby exacerbates immune disorders and activate the inflammatory pathway [47].

The present study includes the following pronounced strengths. First, the samples used were gathered across populations with the same European ancestries, which minimized stratification bias. Second, we used the publicly available GWAS datasets with the largest sample sizes hitherto for both the exposure and outcome datasets, and F-statistics were also large enough to control weak instrumental bias. Third, a range of pleiotropy assessment and sensitivity analyses relaxed the IV assumptions and supported the robustness of our MR findings. However, we must pay attention to several limitations. Firstly, there may have been some participant overlap in exposure and outcomes, which would reduce the data quality. Secondly, the diagnoses of some cases were based on self-reported condition collected through questionnaires, which is possible to lead to misclassification. Lastly, only populations of European ancestry were incorporated into our MR analysis, the findings need to be verified in populations with other ancestries.

## Conclusion

Using a bidirectional MR approach, our results provided suggestive evidence that genetically predicted insomnia was associated with an increased risk of allergic disease, asthma, moderate-severe asthma, and adult-onset asthma. Improving sleep quality may be one of the cornerstones in the prevention of allergic disease and asthma. Conversely, no evidence supported that genetically predicted allergic disease, asthma, or moderate-severe asthma was associated with the risk of insomnia. In future, replication of these findings using even larger GWAS are required.

## Supplementary Information

**Additional file 1**: **Table S1.** Detailed information on data sources in the present study. **Table S4.** Detailed information of Mendelian randomization analysis for insomnia and allergy disease, asthma and its phenotypes. **Table S5.** Detailed information of Mendelian randomization analysis for allergy disease, asthma, and its phenotype and insomnia; **Figure S1.** Funnel plots for MR analyses of the causal effect of insomnia on allergic disease, asthma and it’s phenotypes. **Figures S2–S6.** Leave-one-out sensitivity based on IVW model for insomnia on allergic disease, asthma and its phenotypes. **Figures S7–S11.** Forest plot for the association between insomnia and allergic disease, asthma, its phenotypes. **Figure S12.** Funnel plots for MR analyses of the causal effect of allergic disease, asthma, and moderate–severe asthma on insomnia. **Figures S13–S15.** Leave-one-out sensitivity based on IVW model for allergic disease, asthma, moderate-severe asthma on insomnia. **Figure S16–S18.** Forest plot for the association between allergic disease, asthma, moderate-severe asthma and insomnia.

**Additional file 2**: **Table S2.** Detailed information on genetic instruments. **Table S3.** SNPs used in current Mendelian randomization analysis.

## Data Availability

All data used in the present study were obtained from genome-wide association study summary statistics which were publicly released by genetic consortia. Data can be obtained by a reasonable request to the corresponding author. All datasets generated for this study are included in the article/Additional files.

## Abbreviations

MR: Mendelian randomization
SNPs: Single nucleotide polymorphisms
IVs: Instrumental variables
GWAS: Genome-wide association studies
LD: Linkage disequilibrium between loci
GASP: The Genetics of Asthma Severity and Phenotype study
U-BIOPRED: The Unbiased Biomarker Prediction of respiratory diseases outcomes project
IVW: The inverse variance weighted
MR-PRESSO: Mendelian Randomization Pleiotropy RESidual Sum and Outlier
OR: Odds ratio
CIs: Confidence intervals
FEV_1_: Forced expiratory volume in one second
TH1: T helper 1 cell

## References

[1] Buysse DJ (2013). Insomnia. Jama.

[2] Jansen PR, Watanabe K, Stringer S, Skene N, Bryois J, Hammerschlag AR, de Leeuw CA, Benjamins JS, Muñoz-Manchado AB, Nagel M (2019). Genome-wide analysis of insomnia in 1,331,010 individuals identifies new risk loci and functional pathways. Nat Genet.

[3] Levenson JC, Kay DB, Buysse DJ (2015). The pathophysiology of insomnia. Chest.

[4] Javaheri S, Redline S (2017). Insomnia and risk of cardiovascular disease. Chest.

[5] Gebara MA, Siripong N, DiNapoli EA, Maree RD, Germain A, Reynolds CF, Kasckow JW, Weiss PM, Karp JF (2018). Effect of insomnia treatments on depression: a systematic review and meta-analysis. Depress Anxiety.

[6] Platts-Mills TA (2015). The allergy epidemics: 1870–2010. J Allergy Clin Immunol.

[7] GBD 2016 Disease and Injury Incidence and Prevalence Collaborators. Global, regional, and national incidence, prevalence, and years lived with disability for 328 diseases and injuries for 195 countries, 1990–2016: a systematic analysis for the Global Burden of Disease Study 2016. Lancet. 2017;390:1211–59. 10.1016/S0140-6736(17)32154-2 PMC5605509 28919117

[8] Brumpton B, Mai XM, Langhammer A, Laugsand LE, Janszky I, Strand LB (2017). Prospective study of insomnia and incident asthma in adults: the HUNT study. Eur Respir J.

[9] Chang YS, Chiang BL (2018). Sleep disorders and atopic dermatitis: a 2-way street?. J Allergy Clin Immunol.

[10] Kavanagh J, Jackson DJ, Kent BD (2018). Sleep and asthma. Curr Opin Pulm Med.

[11] Lin YC, Lai CC, Chien CC, Chen CM, Chiang SR, Ho CH, Weng SF, Cheng KC (2017). Is insomnia a risk factor for new-onset asthma? A population-based study in Taiwan. BMJ Open.

[12] Kim DJ, Ha TW, Jung HU, Baek EJ, Lee WJ, Kim HK, Kang JO, Won S, Lim JE, Oh B (2021). Characterisation of insomnia as an environmental risk factor for asthma via Mendelian randomization and gene environment interaction. Sci Rep.

[13] Lawlor DA, Harbord RM, Sterne JA, Timpson N, Davey Smith G (2008). Mendelian randomization: using genes as instruments for making causal inferences in epidemiology. Stat Med.

[14] Glymour MM, Tchetgen Tchetgen EJ, Robins JM (2012). Credible Mendelian randomization studies: approaches for evaluating the instrumental variable assumptions. Am J Epidemiol.

[15] Yuan S, Mason AM, Burgess S, Larsson SC (2021). Genetic liability to insomnia in relation to cardiovascular diseases: a Mendelian randomisation study. Eur J Epidemiol.

[16] Ferreira MA, Vonk JM, Baurecht H, Marenholz I, Tian C, Hoffman JD, Helmer Q, Tillander A, Ullemar V, van Dongen J (2017). Shared genetic origin of asthma, hay fever and eczema elucidates allergic disease biology. Nat Genet.

[17] Sudlow C, Gallacher J, Allen N, Beral V, Burton P, Danesh J, Downey P, Elliott P, Green J, Landray M (2015). UK biobank: an open access resource for identifying the causes of a wide range of complex diseases of middle and old age. PLoS Med.

[18] Zhu Z, Zhu X, Liu CL, Shi H, Shen S, Yang Y, Hasegawa K, Camargo Jr. CA, Liang L. Shared genetics of asthma and mental health disorders: a large-scale genome-wide cross-trait analysis. Eur Respir J. 2019;54. 10.1183/13993003.01507-2019 31619474

[19] Shrine N, Portelli MA, John C, Soler Artigas M, Bennett N, Hall R, Lewis J, Henry AP, Billington CK, Ahmad A (2019). Moderate-to-severe asthma in individuals of European ancestry: a genome-wide association study. Lancet Respir Med.

[20] Gill D, Efstathiadou A, Cawood K, Tzoulaki I, Dehghan A (2019). Education protects against coronary heart disease and stroke independently of cognitive function: evidence from Mendelian randomization. Int J Epidemiol.

[21] Burgess S, Small DS, Thompson SG (2017). A review of instrumental variable estimators for Mendelian randomization. Stat Methods Med Res.

[22] Burgess S, Dudbridge F, Thompson SG (2016). Combining information on multiple instrumental variables in Mendelian randomization: comparison of allele score and summarized data methods. Stat Med.

[23] Burgess S, Bowden J, Fall T, Ingelsson E, Thompson SG (2017). Sensitivity analyses for robust causal inference from Mendelian randomization analyses with multiple genetic variants. Epidemiology.

[24] Bowden J, Davey Smith G, Haycock PC, Burgess S (2016). Consistent estimation in Mendelian randomization with some invalid instruments using a weighted median estimator. Genet Epidemiol.

[25] Bowden J, Davey Smith G, Burgess S (2015). Mendelian randomization with invalid instruments: effect estimation and bias detection through Egger regression. Int J Epidemiol.

[26] Burgess S, Thompson SG (2017). Interpreting findings from Mendelian randomization using the MR-Egger method. Eur J Epidemiol.

[27] Wu PF, Zhang XH, Zhou P, Yin R, Zhou XT, Zhang W (2021). Growth differentiation factor 15 is associated with Alzheimer’s disease risk. Front Genet.

[28] Bowden J, Del Greco MF, Minelli C, Davey Smith G, Sheehan NA, Thompson JR (2016). Assessing the suitability of summary data for two-sample Mendelian randomization analyses using MR-Egger regression: the role of the I2 statistic. Int J Epidemiol.

[29] Zhang J, Lam SP, Li SX, Yu MW, Li AM, Ma RC, Kong AP, Wing YK (2012). Long-term outcomes and predictors of chronic insomnia: a prospective study in Hong Kong Chinese adults. Sleep Med.

[30] Maidstone RJ, Turner J, Vetter C, Dashti HS, Saxena R, Scheer F, Shea SA, Kyle SD, Lawlor DA, Loudon ASI (2021). Night shift work is associated with an increased risk of asthma. Thorax.

[31] Meltzer LJ, Faino A, Szefler SJ, Strand M, Gelfand EW, Beebe DW (2015). Experimentally manipulated sleep duration in adolescents with asthma: feasibility and preliminary findings. Pediatr Pulmonol.

[32] Zhang S, Liu X, Kim JS, Ouyang F, Wang B, Li Z, Tang G, Liu X, Xu X, Pongracic JA, Wang X (2011). Association between short sleep duration and the risk of sensitization to food and aero allergens in rural Chinese adolescents. Clin Exp Allergy.

[33] Chen Y, Yang Q, Zhao K, Wu Z, Shen X, Li S (2021). Associations of sleep characteristics with atopic disease: a cross-sectional study among Chinese adolescents. Allergy Asthma Clin Immunol.

[34] Kozyrskyj AL, Kendall GE, Zubrick SR, Newnham JP, Sly PD (2009). Frequent nocturnal awakening in early life is associated with nonatopic asthma in children. Eur Respir J.

[35] Carter KA, Hathaway NE, Lettieri CF (2014). Common sleep disorders in children. Am Fam Physician.

[36] Chu S, Wu Z, Wu Z, Wu J, Qian Y (2021). Association between insomnia and migraine risk: a case-control and bidirectional Mendelian randomization study. Pharmgenom Pers Med.

[37] Besedovsky L, Lange T, Born J (2012). Sleep and immune function. Pflugers Arch.

[38] Besedovsky L, Lange T, Haack M (2019). The sleep-immune crosstalk in health and disease. Physiol Rev.

[39] Bollinger T, Bollinger A, Skrum L, Dimitrov S, Lange T, Solbach W (2009). Sleep-dependent activity of T cells and regulatory T cells. Clin Exp Immunol.

[40] Slavich GM, Irwin MR (2014). From stress to inflammation and major depressive disorder: a social signal transduction theory of depression. Psychol Bull.

[41] Irwin MR, Cole SW (2011). Reciprocal regulation of the neural and innate immune systems. Nat Rev Immunol.

[42] Chang YS, Chiang BL (2016). Mechanism of sleep disturbance in children with atopic dermatitis and the role of the circadian rhythm and melatonin. Int J Mol Sci.

[43] Sakami S, Ishikawa T, Kawakami N, Haratani T, Fukui A, Kobayashi F, Fujita O, Araki S, Kawamura N (2002). Coemergence of insomnia and a shift in the Th1/Th2 balance toward Th2 dominance. NeuroImmunoModulation.

[44] Papi A, Brightling C, Pedersen SE, Reddel HK (2018). Asthma. Lancet.

[45] Kubo M (2017). Innate and adaptive type 2 immunity in lung allergic inflammation. Immunol Rev.

[46] Cho JH, Bhutani S, Kim CH, Irwin MR (2021). Anti-inflammatory effects of melatonin: a systematic review and meta-analysis of clinical trials. Brain Behav Immun.

[47] Higuchi S, Nagafuchi Y, Lee SI, Harada T (2014). Influence of light at night on melatonin suppression in children. J Clin Endocrinol Metab.

