# Supplementary material for: Exploring genetic association of insomnia with allergic disease and asthma: a bidirectional Mendelian randomization study
Source: Respir Res. 2022 Apr 7;23:84. doi: 10.1186/s12931-022-02009-6 (PMC8991606; doi:10.1186/s12931-022-02009-6)
Supplement: Supplementary file 1 — Additional file 1: Table S1. Detailed information on data sources in the present study. Table S4. Detailed information of Mendelian randomization analysis for insomnia and allergy disease, asthma and its phenotypes. Table S5. Detailed information of Mendelian randomization analysis for allergy disease, asthma, and its phenotype and insomnia; Figure S1. Funnel plots for MR analyses of the causal effect of insomnia on allergic disease, asthma and it’s phenotypes. Figures S2–S6. Leave-one-out sensitivity based on IVW model for insomnia on allergic disease, asthma and its phenotypes. Figures S7–S11. Forest plot for the association between insomnia and allergic disease, asthma, its phenotypes. Figure S12. Funnel plots for MR analyses of the causal effect of allergic disease, asthma, and moderate–severe asthma on insomnia. Figures S13–S15. Leave-one-out sensitivity based on IVW model for allergic disease, asthma, moderate-severe asthma on insomnia. Figure S16–S18. Forest plot for the association between allergic disease, asthma, moderate-severe asthma and insomnia. [file 12931_2022_2009_MOESM1_ESM.docx]

**Exploring Genetic Association of Insomnia with Allergic Disease and Asthma: A Bidirectional Mendelian Randomization Study**

Rong Li^a^, Yiting Chen^a^, Anda Zhao^b^, Lili Huang^a^, Zichong Long^a^, Wenhui Kang^a^, Yong Yin^c^, Shilu Tong^a,c,d,e^, Yongmei Guo ^f^*****, Shenghui Li^a,g^*****

^a^ School of Public Health, Shanghai Jiao Tong University School of Medicine, Shanghai, China

^b^ Shanghai Ninth People's Hospital, Shanghai Jiao Tong University School of Medicine, Shanghai, China

^c^ Shanghai Children's Medical Center, Shanghai Jiao Tong University School of Medicine, Shanghai, China

^d^ School of Public Health, Institute of Environment and Population Health, Anhui Medical University, Hefei, China

^e^ School of Public Health and Social Work, Queensland University of Technology, Brisbane, Australia

^f^ Department of Neurology, Shuguang Hospital Affiliated to Shanghai University of traditional Chinese Medicine, Shanghai, China

^g^ MOE-Shanghai Key Laboratory of Children’s Environmental Health, Shanghai Jiao Tong University School of Medicine, Shanghai, China

***Correspondence:**

Shenghui Li, MD, Ph.D, School of Public Health, Shanghai Jiao Tong University, 227 South Chongqing Road, Huangpu District, Shanghai, 200025, China. Tel: +86-21-63846590; E-mail: [lsh9907@163.com](mailto:lsh9907@163.com), [submission9907@163.com](mailto:submission9907@163.com)

Yongmei Guo, MD, Ph.D, Shuguang Hospital Affiliated to Shanghai University of traditional Chinese Medicine, 528 Zhangheng Road, Pudong District, Shanghai, 201203, China. Tel: +86-21-53821650; E-mail: [yongmeiguo@126.com](file:///C:\Users\90582\Downloads\RERE-D-21-01050\yongmeiguo@126.com)

**Additional file 1**

**Table S1:** Detailed information on data sources in the present study

**Table S4:** Detailed information of Mendelian randomization analysis for genetically predicted insomnia and allergy disease, asthma and its phenotypes

**Table S5:** Detailed information of Mendelian randomization analysis for allergy disease, asthma, and its phenotype and insomnia

**Figure S1:** Funnel plots for MR analyses of the causal effect of insomnia on allergic disease, asthma and its phenotypes

**Figure S2:** Leave-one-out sensitivity based on IVW model for insomnia on allergic disease

**Figure S3:** Leave-one-out sensitivity based on IVW model for insomnia on asthma

**Figure S4:** Leave-one-out sensitivity based on IVW model for insomnia on moderate-severe asthma

**Figure S5:** Leave-one-out sensitivity based on IVW model for insomnia on adult-onset asthma

**Figure S6:** Leave-one-out sensitivity based on IVW model for insomnia on childhood asthma

**Figure S7:** Forest plot for the association between insomnia and allergic disease

**Figure S8:** Forest plot for the association between insomnia and asthma

**Figure S9:** Forest plot for the association between insomnia and moderate-severe asthma

**Figure S10:** Forest plot for the association between insomnia and adult-onset asthma

**Figure S11:** Forest plot for the association between insomnia and childhood asthma.

**Figure S12:** Funnel plots for MR analyses of the causal effect of allergic disease, asthma, and moderate–severe asthma on insomnia

**Figure S13:** Leave-one-out sensitivity based on IVW model for allergic disease on insomnia

**Figure S14:** Leave-one-out sensitivity based on IVW model for asthma on insomnia

**Figure S15:** Leave-one-out sensitivity based on IVW model for moderate-severe asthma on insomnia

**Figure S16:** Forest plot for the association between allergic disease and insomnia

**Figure S17:** Forest plot for the association between asthma and insomnia

**Figure S18:** Forest plot for the association between moderate-severe and insomnia

| **Table S1** Detailed information on data sources in the present study | | | | | | |  |
| --- | --- | --- | --- | --- | --- | --- | --- |
| **Traits** | **PMID** | **Ancestry** | **Sample size** | **Cases** | **Controls** | **Sample source** | |
| **Insomnia** | 30804565 | European | 1,331,010 | 397,959 | 933,051 | 23andMe (944,477) and UK Biobank (386,533) | |
| **Allergic disease** | 29083406 | European | 360,838 | 180,129 | 180,709 | 13 studies; UK Biobank (138,354), 23andMe (118,269), GERA (51,218), CATSS (11,068), NTR (10,242), LifeLines (8,560), TWINGENE (5,517), ALSPAC (4,964), SALTY (4,062), GENEVA (2,633), AAGC (2,435), GENUFAD-SHIP-1 (1,781), GENUFAD-SHIP-2 (1,735) | |
| **Asthma** | 31619474 | European | 394,283 | 46,802 | 347,481 | UK Biobank (394,283) | |
| **Moderate–severe asthma** | 30552067 | European | 30,810 | 5,135 | 25,675 | GASP (1,858), U-BIOPRED (356) and UK Biobank (28,596) | |
| **Adult-onset asthma** | 31619474 | European | 369,777 | 22,296 | 347,481 | UK Biobank (369,777) | |
| **Childhood asthma** | 31619474 | European | 357,157 | 9,676 | 347,481 | UK Biobank (357,157) | |

| **Table S4** Detailed information of Mendelian randomization analysis for insomnia and allergy disease, asthma and its phenotypes | | | | | |
| --- | --- | --- | --- | --- | --- |
|  |  | **Inverse Variance Weighted (IVW)** | | **Weighted median** | **MR Egger** |
|  |  | **Multiplicative random effect** | **Fixed effect** |  |  |
| **Allergic disease** | ***OR* (95% *CI*)** | 1.054 (1.031,1.078) | 1.054 (1.032,1.077) | 1.041 (1.008,1.075) | 1.011 (0.923,1.109) |
|  | **Beta ± SE** | 0.053 ± 0.011 | 0.053 ± 0.011 | 0.040 ± 0.016 | 0.011 ± 0047 |
|  | ***P*-value** | 3.817×10^-06^ | 9.461×10^-07^ | 1.420×10^-02^ | 8.074×10^-01^ |
| **Asthma** | ***OR* (95% *CI*)** | 1.043 (1.010,1.077) | 1.043 (1.013,1.074) | 1.043 (0.997,1.091) | 0.960 (0.858,1.075) |
|  | **Beta ± SE** | 0.042 ± 0.016 | 0.042 ± 0.015 | 0.042 ± 0.023 | -0.041 ± 0.057 |
|  | ***P*-value** | 9.811×10^-03^ | 4.836×10^-03^ | 6.770×10^-02^ | 4.815×10^-01^ |
| **Moderate–severe asthma** | ***OR* (95% *CI*)** | 1.168 (1.069,1.277) | 1.168 (1.072,1.273) | 1.165 (1.028,1.320) | 1.784 (1.188,2.680) |
|  | **Beta ± SE** | 0.156 ± 0.045 | 0.156 ± 0.044 | 0.153 ± 0.064 | 0.579 ± 0.207 |
|  | ***P*-value** | 6.234×10^-04^ | 4.022×10^-04^ | 1.658×10^-02^ | 5.847×10^-03^ |
| **Adult-onset asthma** | ***OR* (95% *CI*)** | 1.086 (1.037,1.138) | 1.086 (1.041,1.133) | 1.091 (1.022,1.165) | 0.935 (0.795,1.100) |
|  | **Beta ± SE** | 0.083 ± 0.024 | 0.083 ± 0.021 | 0.087 ± 0.033 | -0.067 ± 0.083 |
|  | ***P*-value** | 4.922×10^-04^ | 1.188×10^-04^ | 8.982×10^-03^ | 4.194×10^-01^ |
| **Childhood asthma** | ***OR* (95% *CI*)** | 0.982 (0.915,1.053) | 0.982 (0.919,1.048) | 1.008 (0.907,1.120) | 0.944 (0.738,1.207) |
|  | **Beta ± SE** | -0.019 ± 0.036 | -0.019 ± 0.034 | 0.008 ± 0.054 | 0.058 ± 0.125 |
|  | ***P*-value** | 6.020×10-01 | 5.792×10-01 | 8.874×10-01 | 6.471×10-01 |

*OR*: odds ratio; *CI*: confidence interval; SE: standard error;

| **Table S5** Detailed information of Mendelian randomization analysis for allergy disease, asthma, and its phenotype and insomnia | | | | | |
| --- | --- | --- | --- | --- | --- |
|  |  | **Inverse Variance Weighted (IVW)** | | **Weighted median** | **MR Egger** |
|  |  | **Multiplicative random effect** | **Fixed effect** |  |  |
| **Allergic disease** | ***OR* (95% *CI*)** | 0.989 (0.963,1.017) | 0.989 (0.965,1.014) | 0.987 (0.951,1.025) | 1.005 (0.929,1.088) |
|  | **Beta ± SE** | -0.011 ± 0.014 | -0.011 ± 0.013 | -0.013 ± 0.019 | 0.005 ± 0.040 |
|  | ***P*-value** | 0.448 | 0.405 | 0.508 | 0.896 |
| **Asthma** | ***OR* (95% *CI*)** | 1.008 (0.986,1.032) | 1.008 (0.988,1.030) | 1.003 (0.971,1.036) | 1.005 (0.949,1.064) |
|  | **Beta ± SE** | 0.008 ± 0.012 | 0.008 ± 0.011 | 0.003 ± 0.016 | 0.005 ± 0.029 |
|  | ***P*-value** | 0.470 | 0.430 | 0.873 | 0.875 |
| **Moderate–severe asthma** | ***OR* (95% *CI*)** | 1.001 (0.981,1.021) | 1.001 (0.982,1.020) | 0.989 (0.963,1.016) | 1.028 (0.909,1.163) |
|  | **Beta ± SE** | 0.001 ± 0.010 | 0.001 ± 0.010 | -0.011 ± 0.014 | 0.028 ± 0.063 |
|  | ***P*-value** | 0.930 | 0.928 | 0.422 | 0.668 |

*OR*: odds ratio; *CI*: confidence interval; SE: standard error;
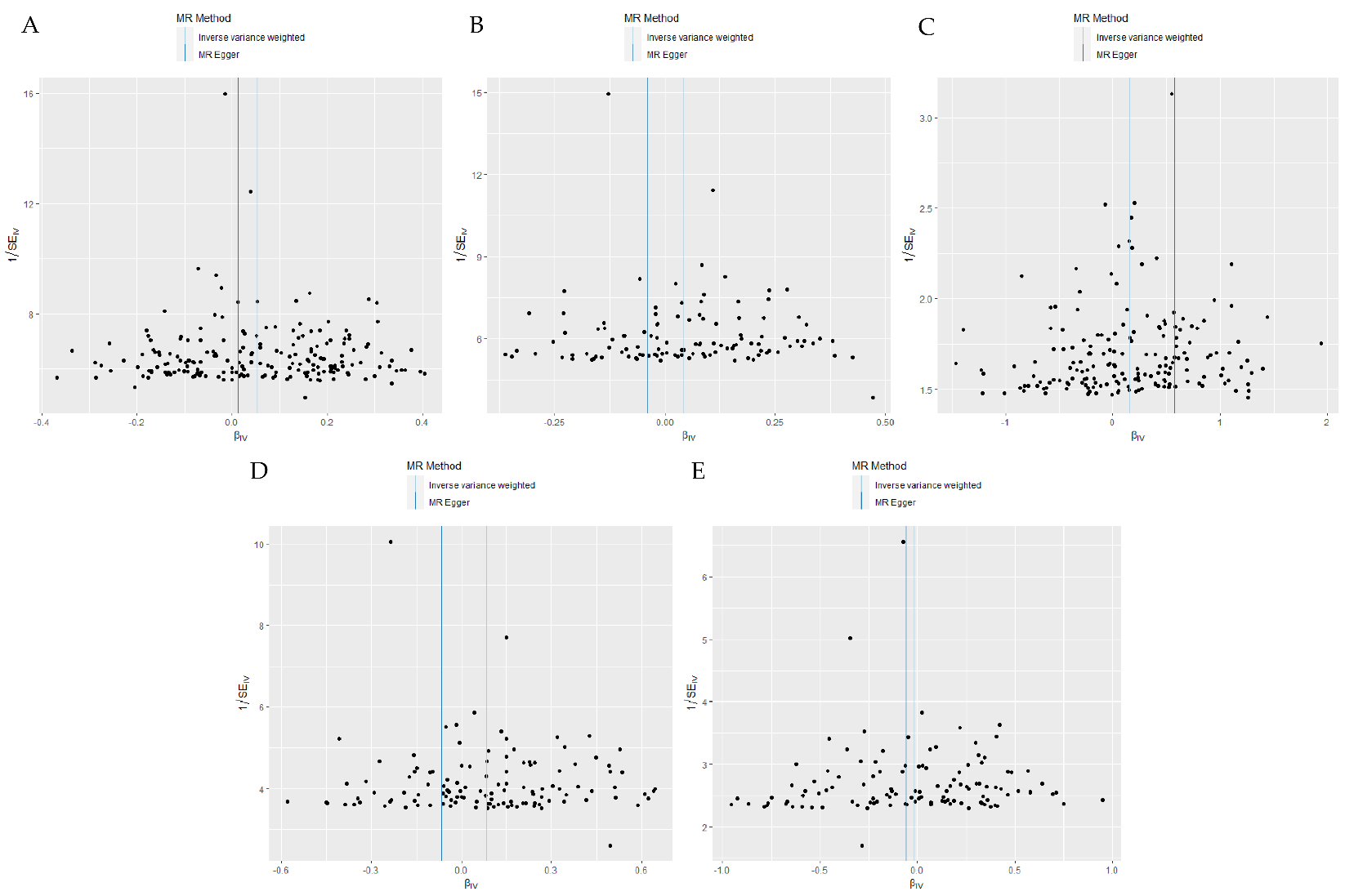


**Figure S1** Funnel plots for MR analyses of the causal effect of insomnia on allergic disease, asthma and it’s phenotypes

A: Allergic disease; B: Asthma; C: Moderate–severe asthma; D: Adult-onset asthma; E: Childhood asthma

**
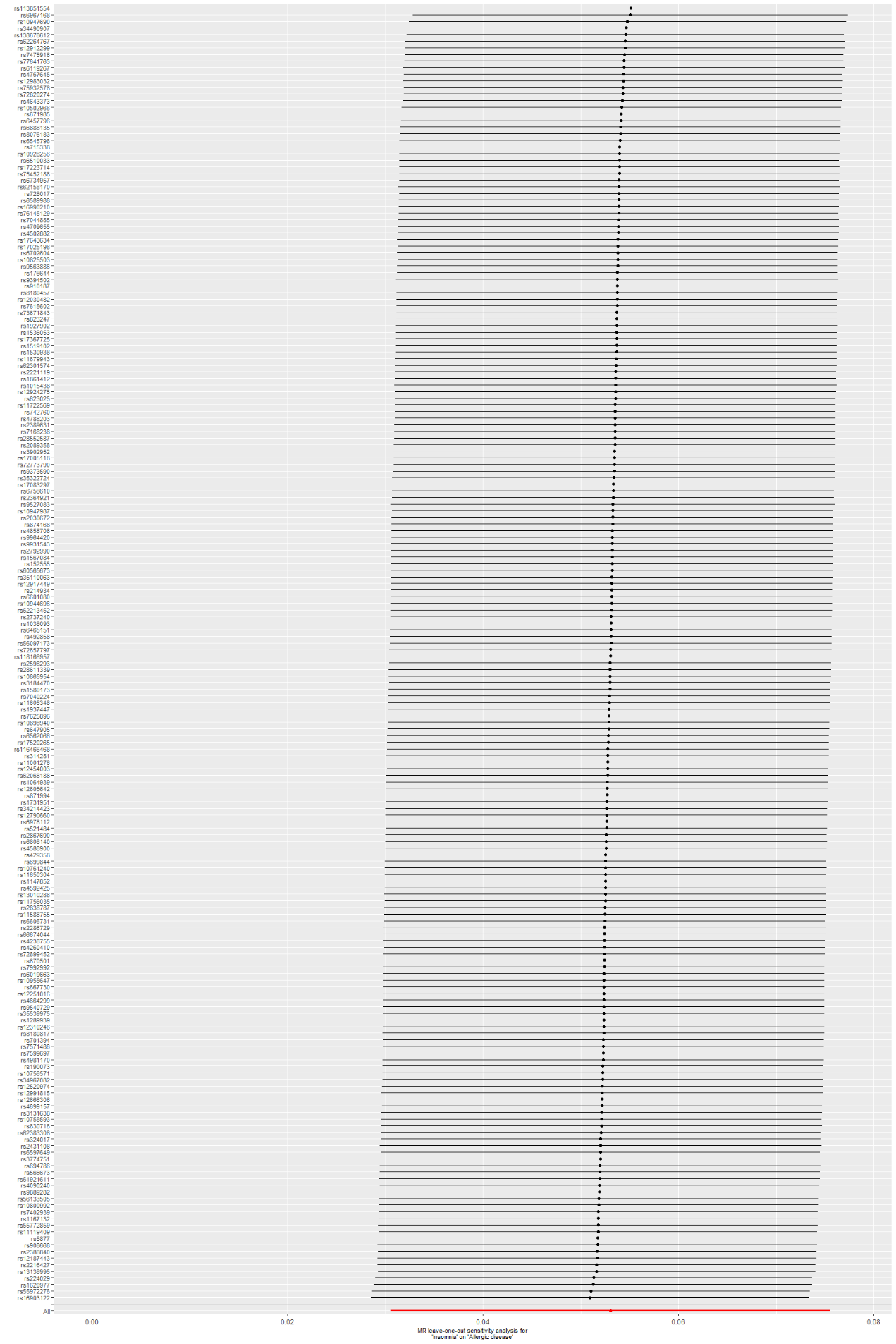
**

**Figure S2** Leave-one-out sensitivity based on IVW model for insomnia on allergic disease

**
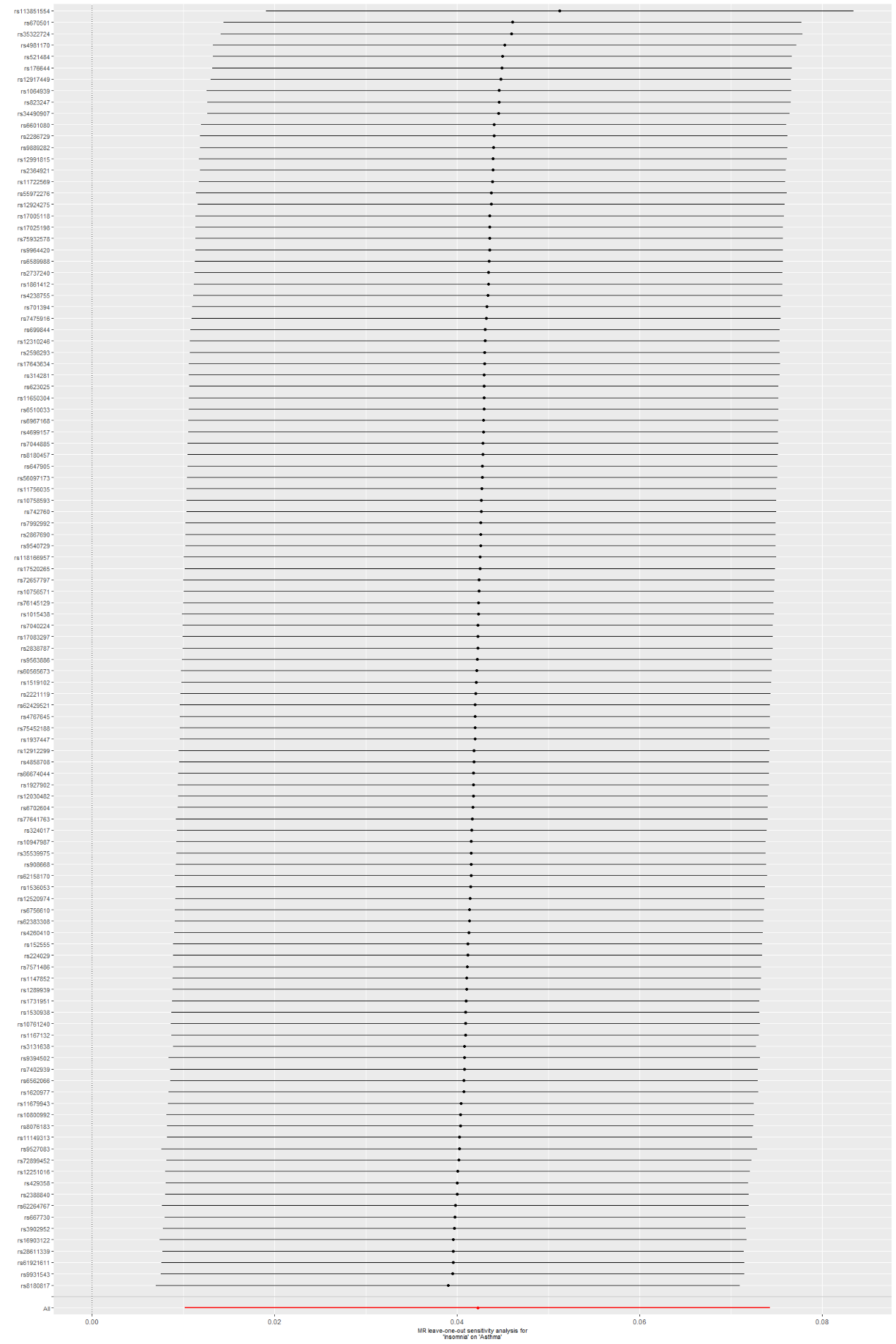
**

**Figure S3** Leave-one-out sensitivity based on IVW model for insomnia on asthma

**
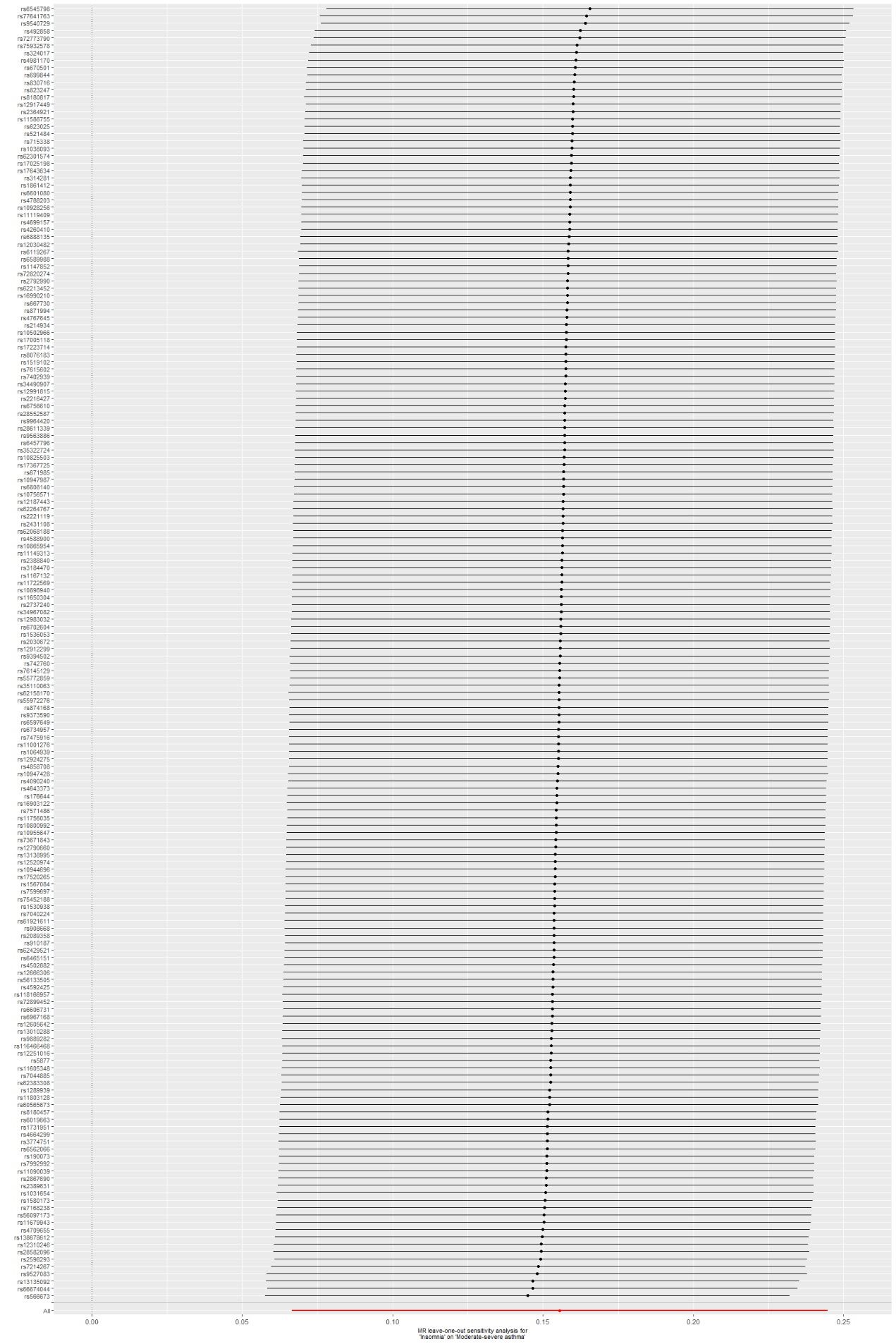
**

**Figure S4** Leave-one-out sensitivity based on IVW model for insomnia on moderate–severe asthma

**
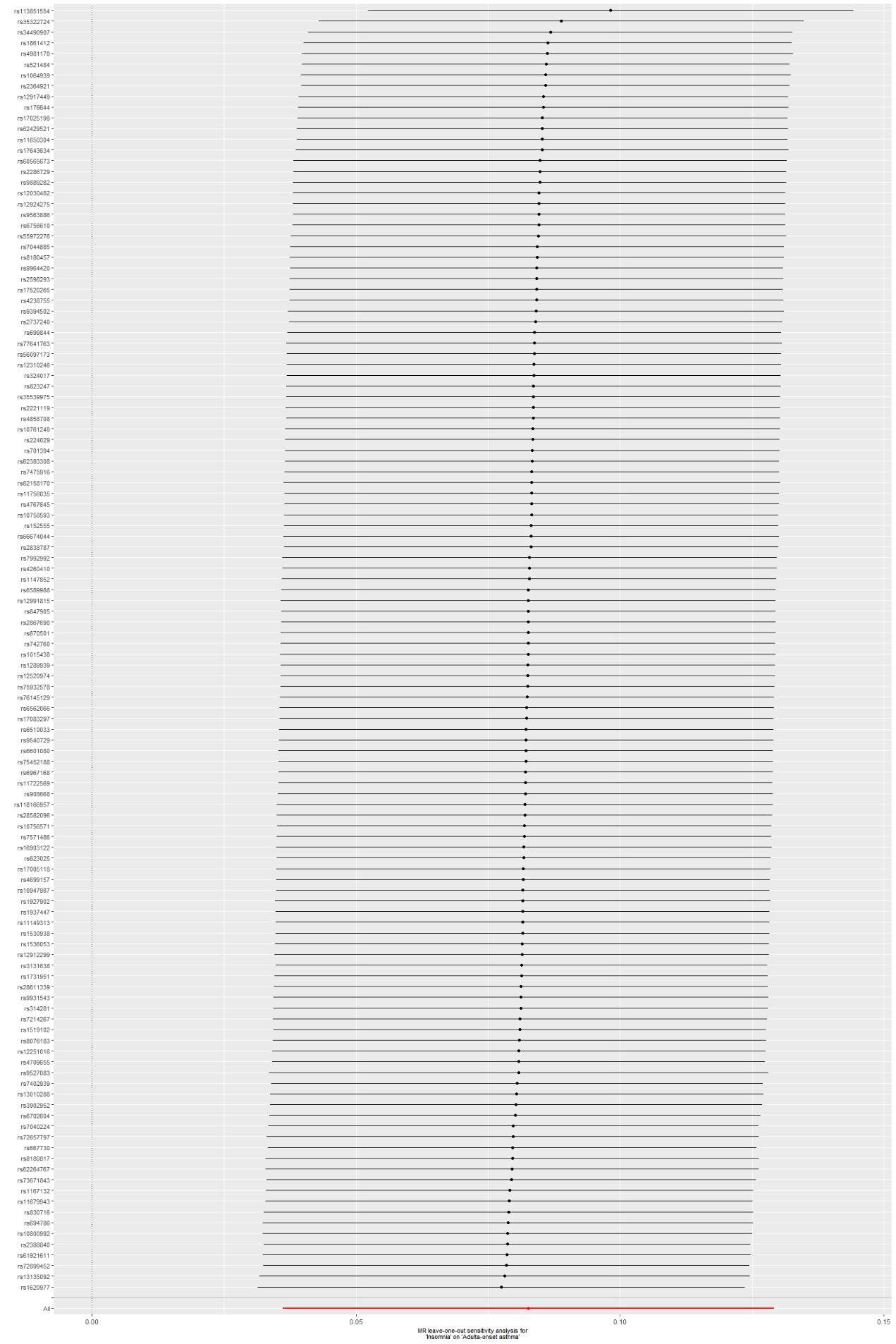
**

**Figure S5** Leave-one-out sensitivity based on IVW model for insomnia on adult-onset asthma

**
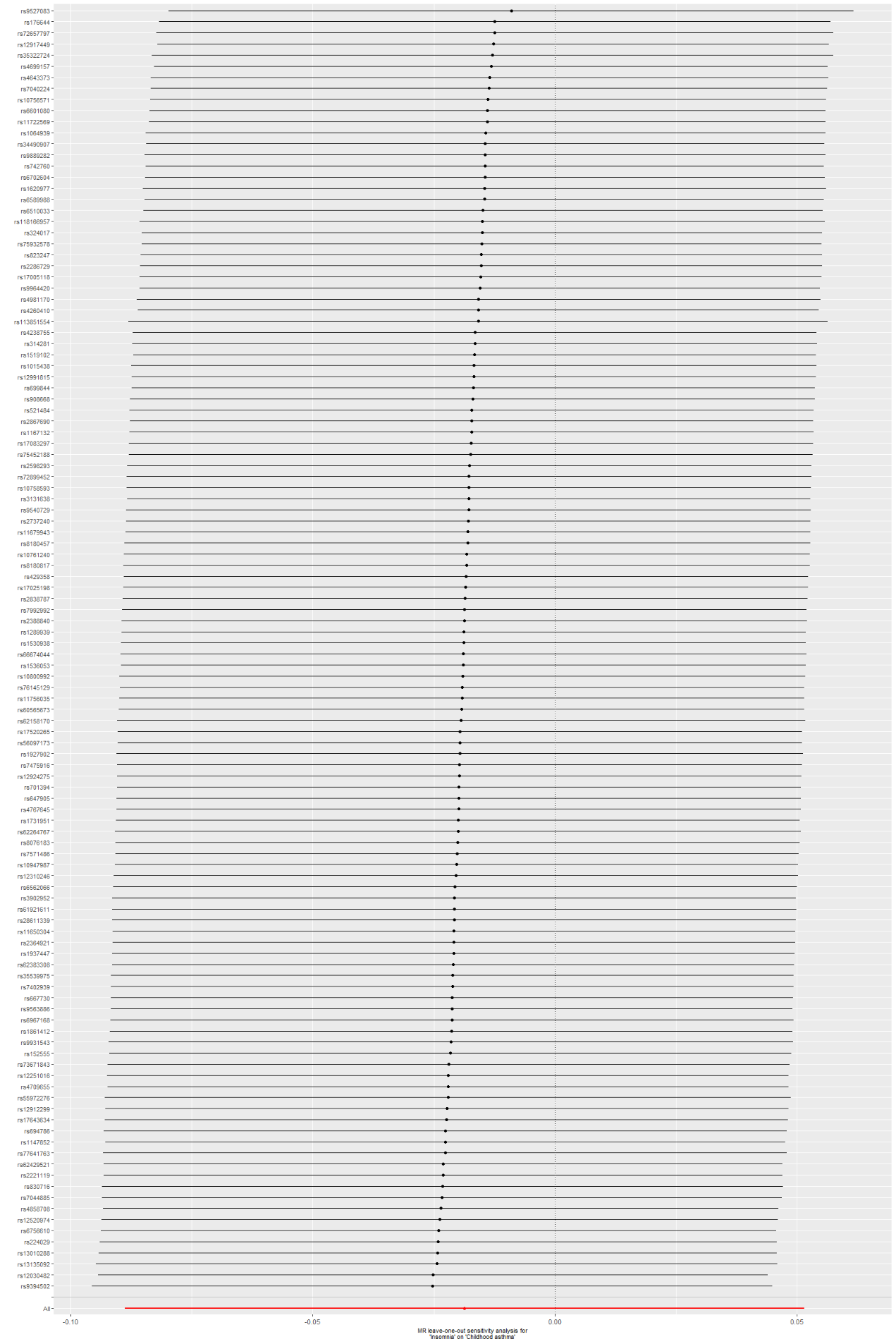
**

**Figure S6** Leave-one-out sensitivity based on IVW model for insomnia on childhood asthma

**
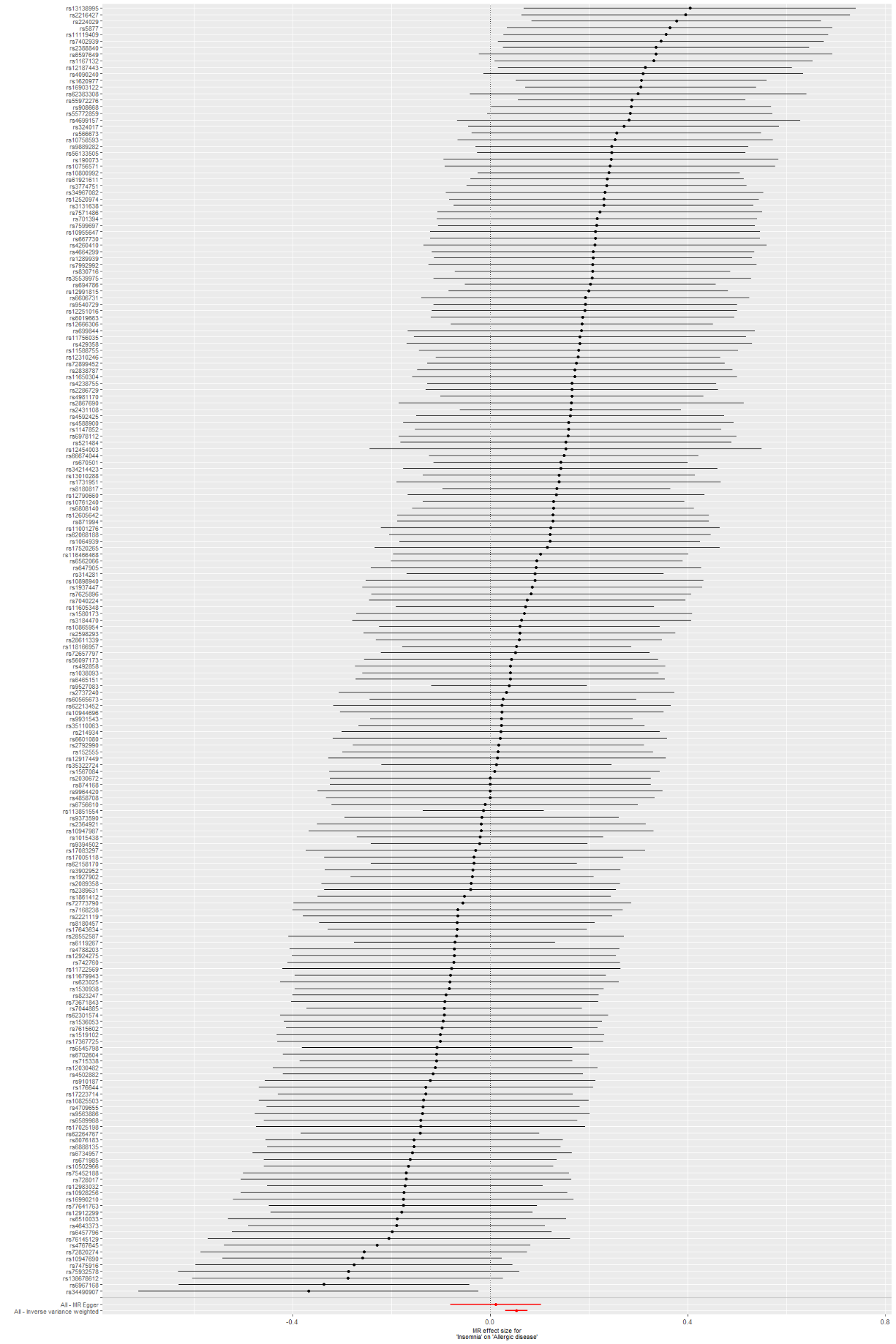
**

**Figure S7** Forest plot for the association between insomnia and allergic disease

**
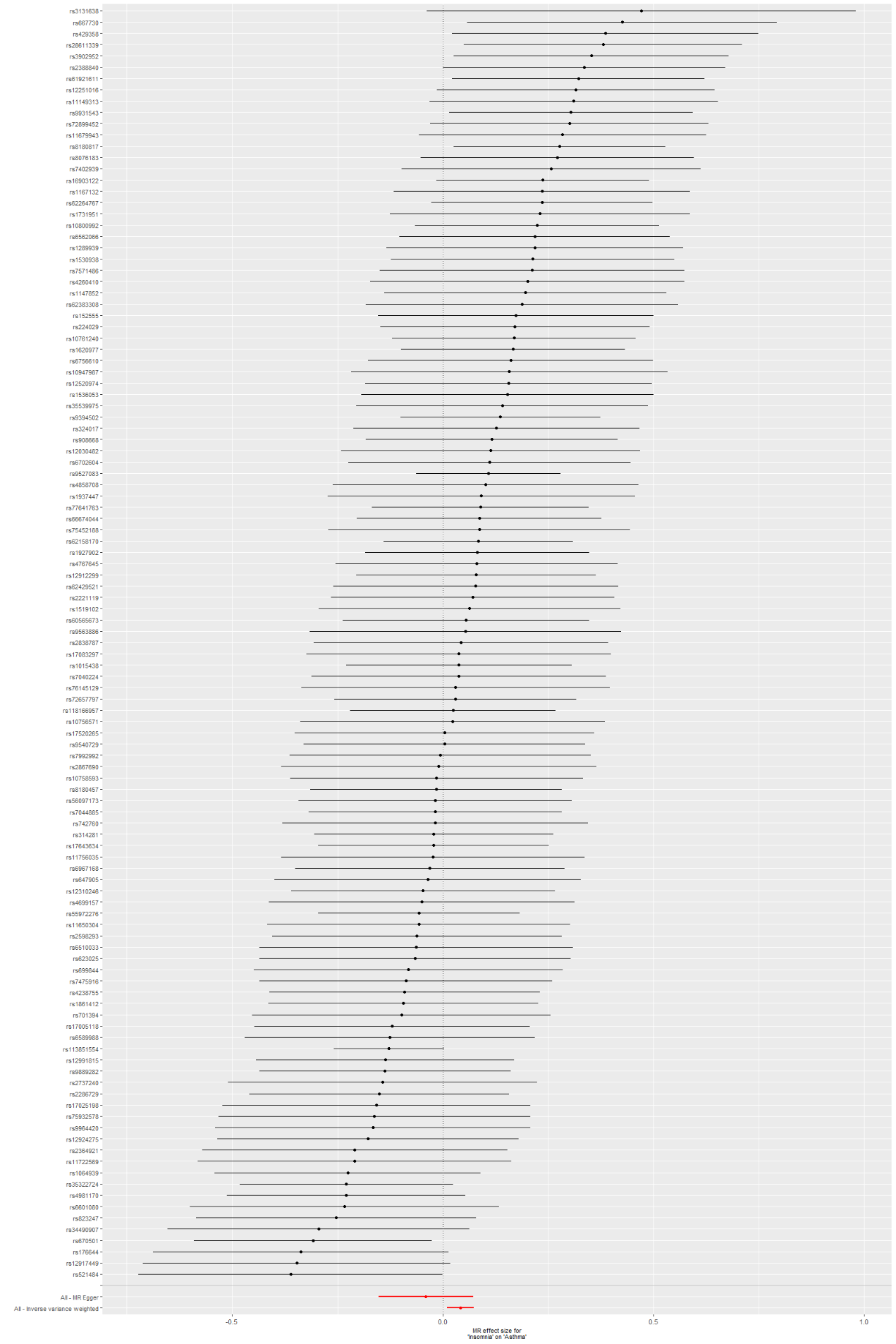
**

**Figure S8** Forest plot for the association between insomnia and asthma

**
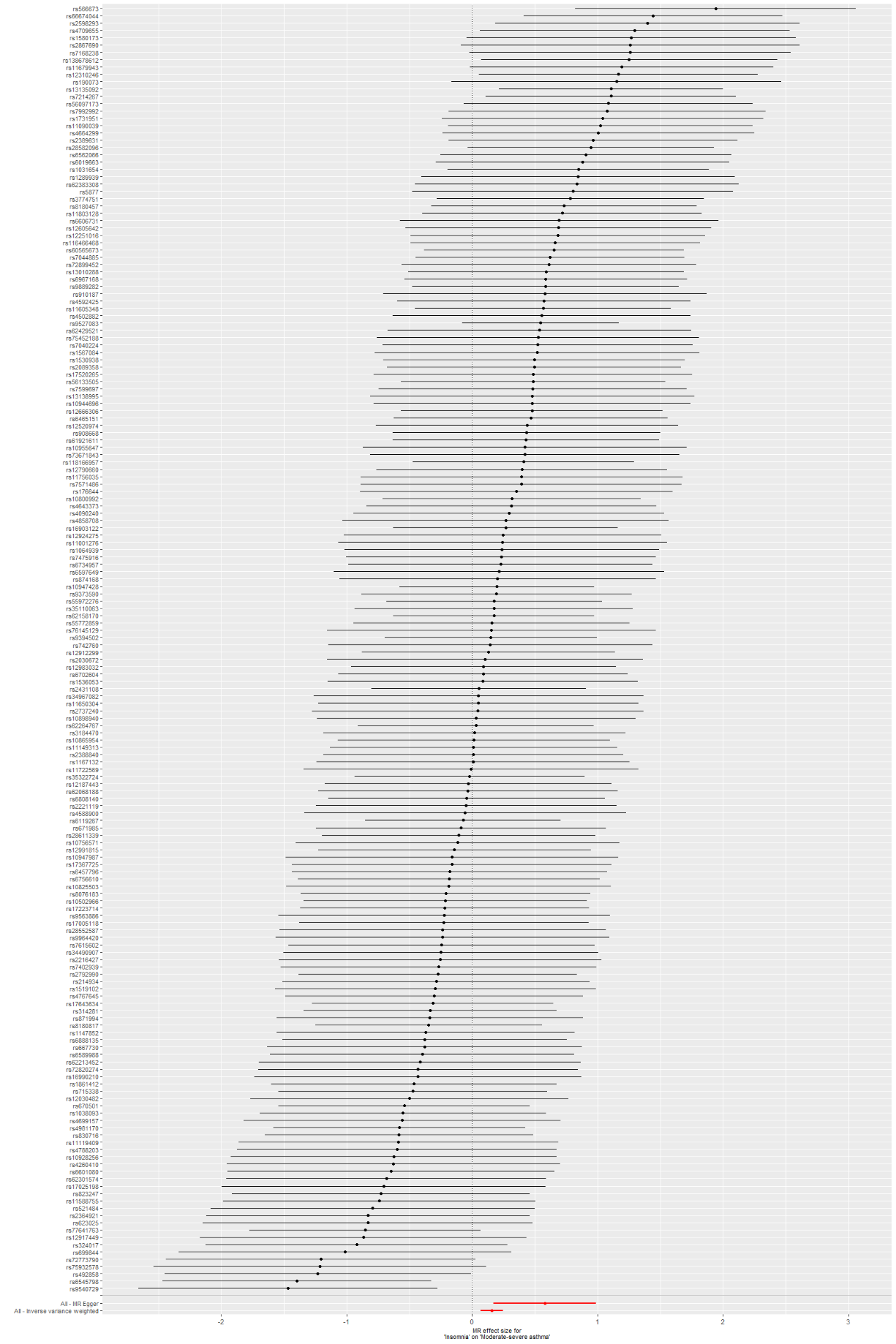
**

**Figure S9** Forest plot for the association between insomnia and moderate–severe asthma

**
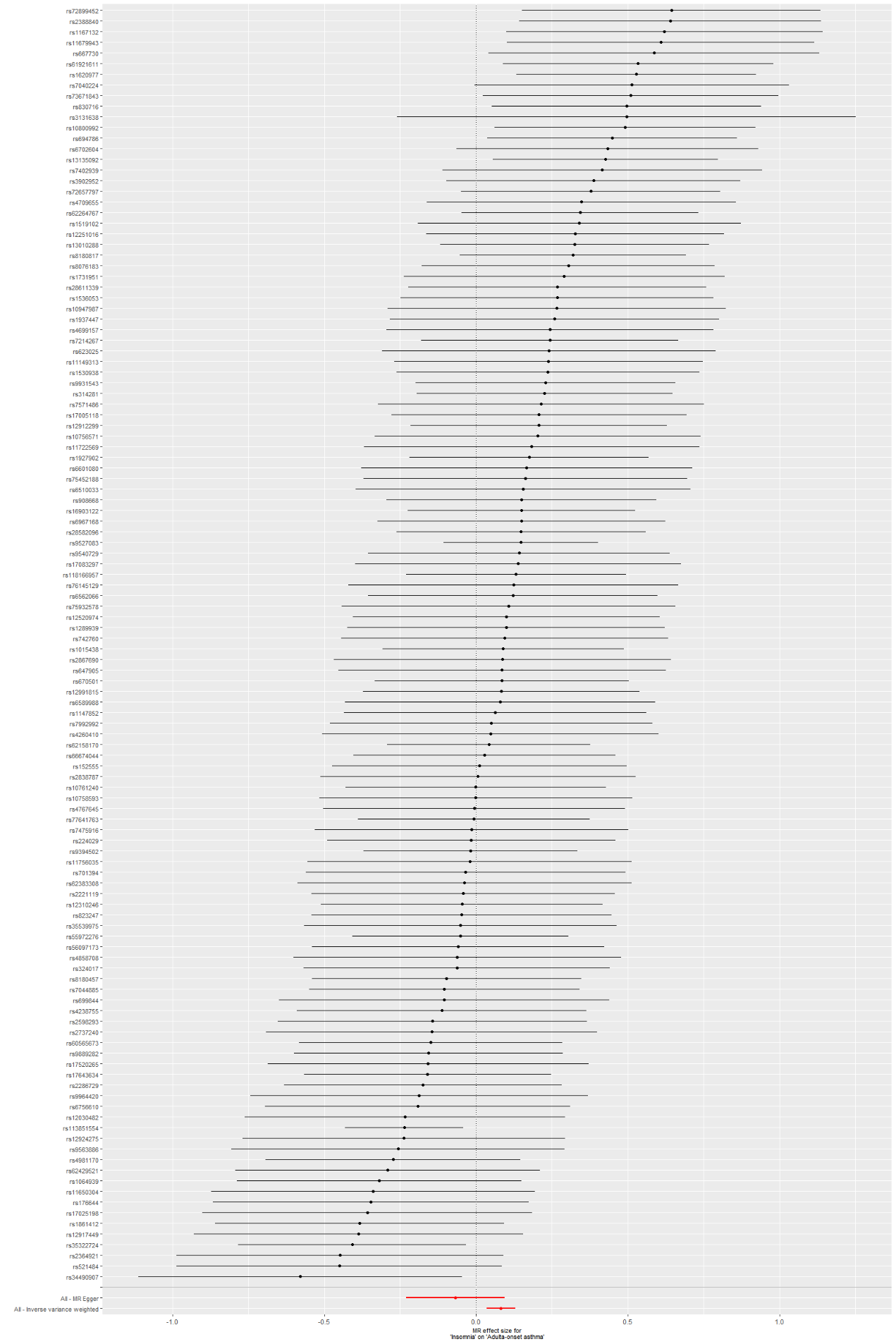
**

**Figure S10** Forest plot for the association between insomnia and adult-onset asthma

**
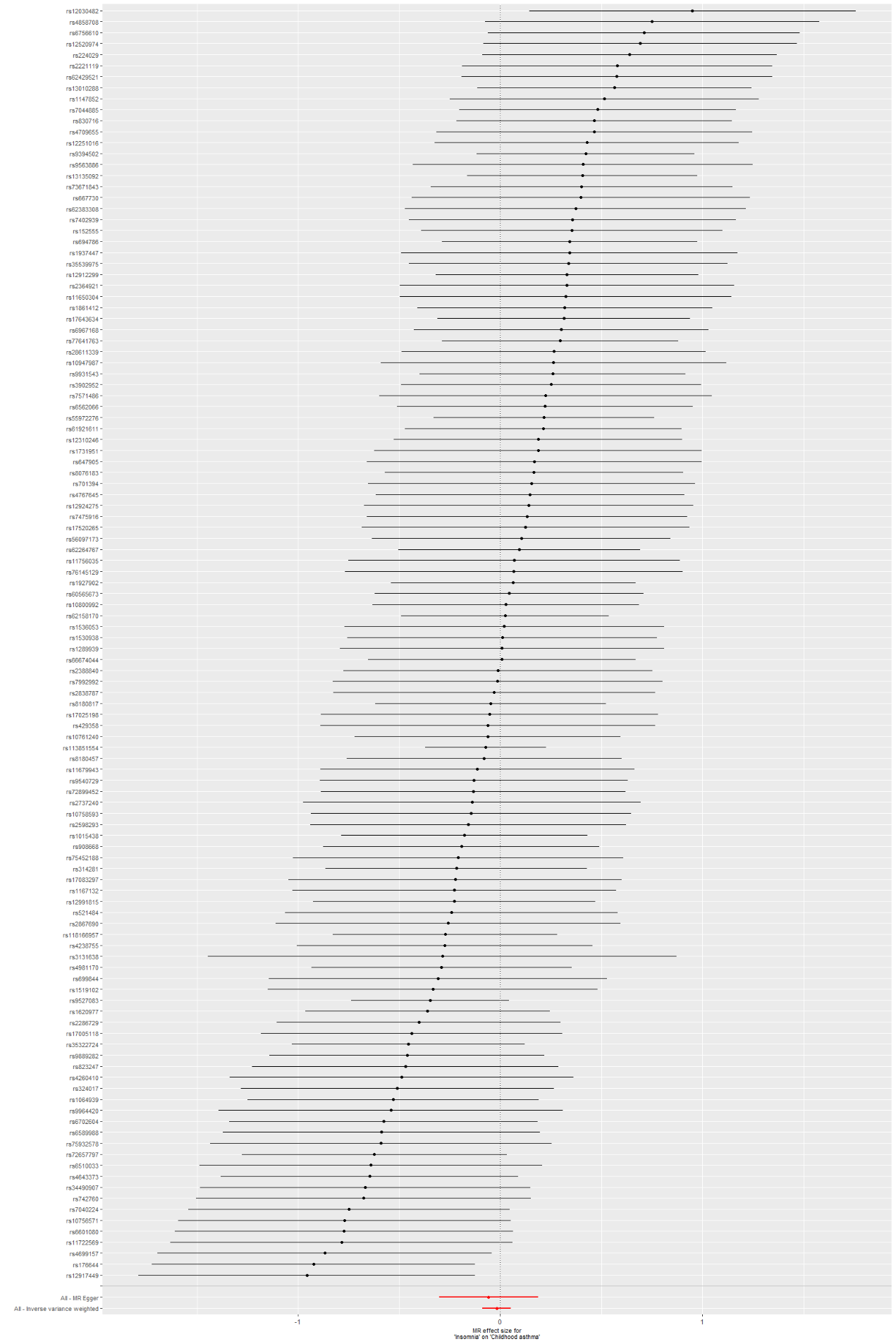
**

**Figure S11** Forest plot for the association between insomnia and childhood asthma


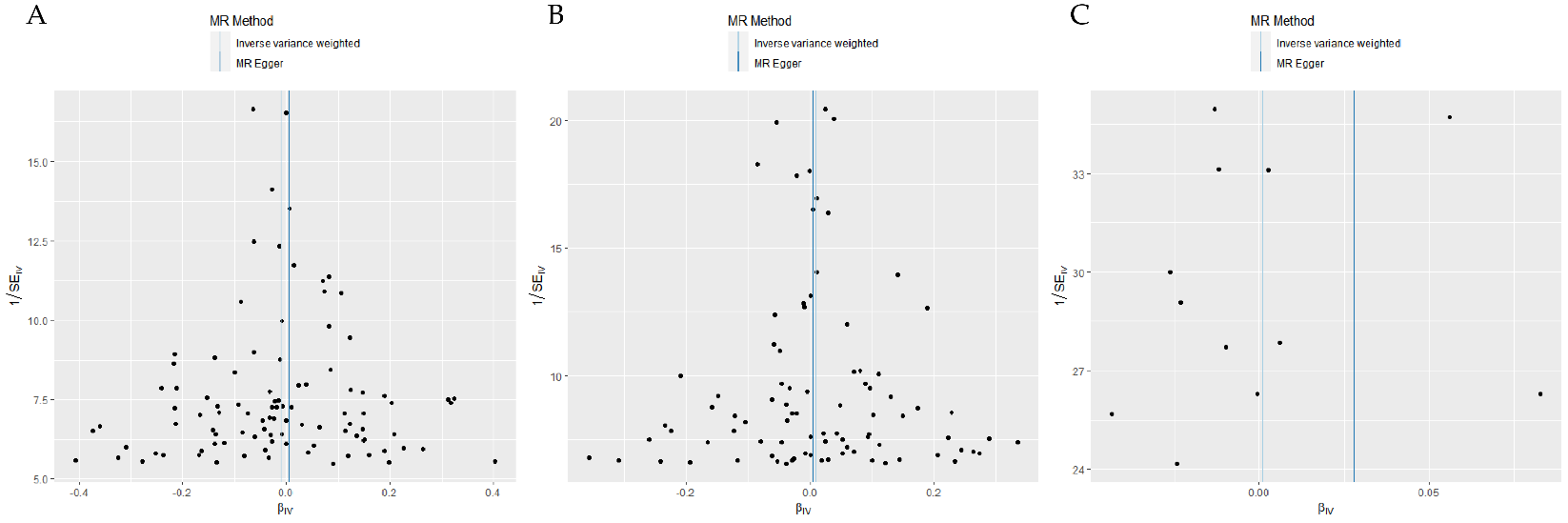


**Figure S12** Funnel plots for MR analyses of the causal effect of allergic disease, asthma, and moderate–severe asthma on insomnia

A: Allergic disease; B: Asthma; C: Moderate–severe asthma


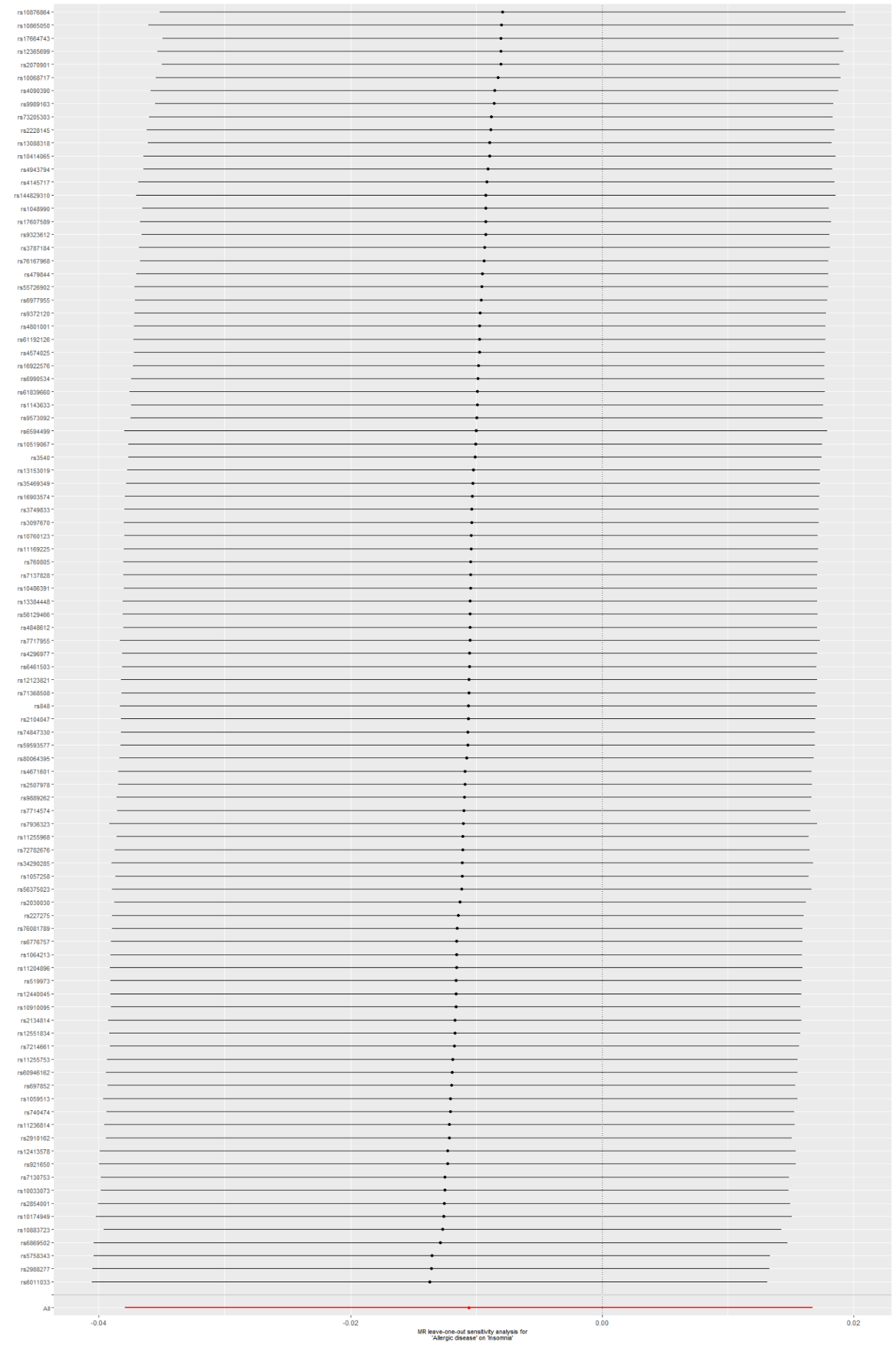


**Figure S13** Leave-one-out sensitivity based on IVW model for allergic disease on insomnia

**
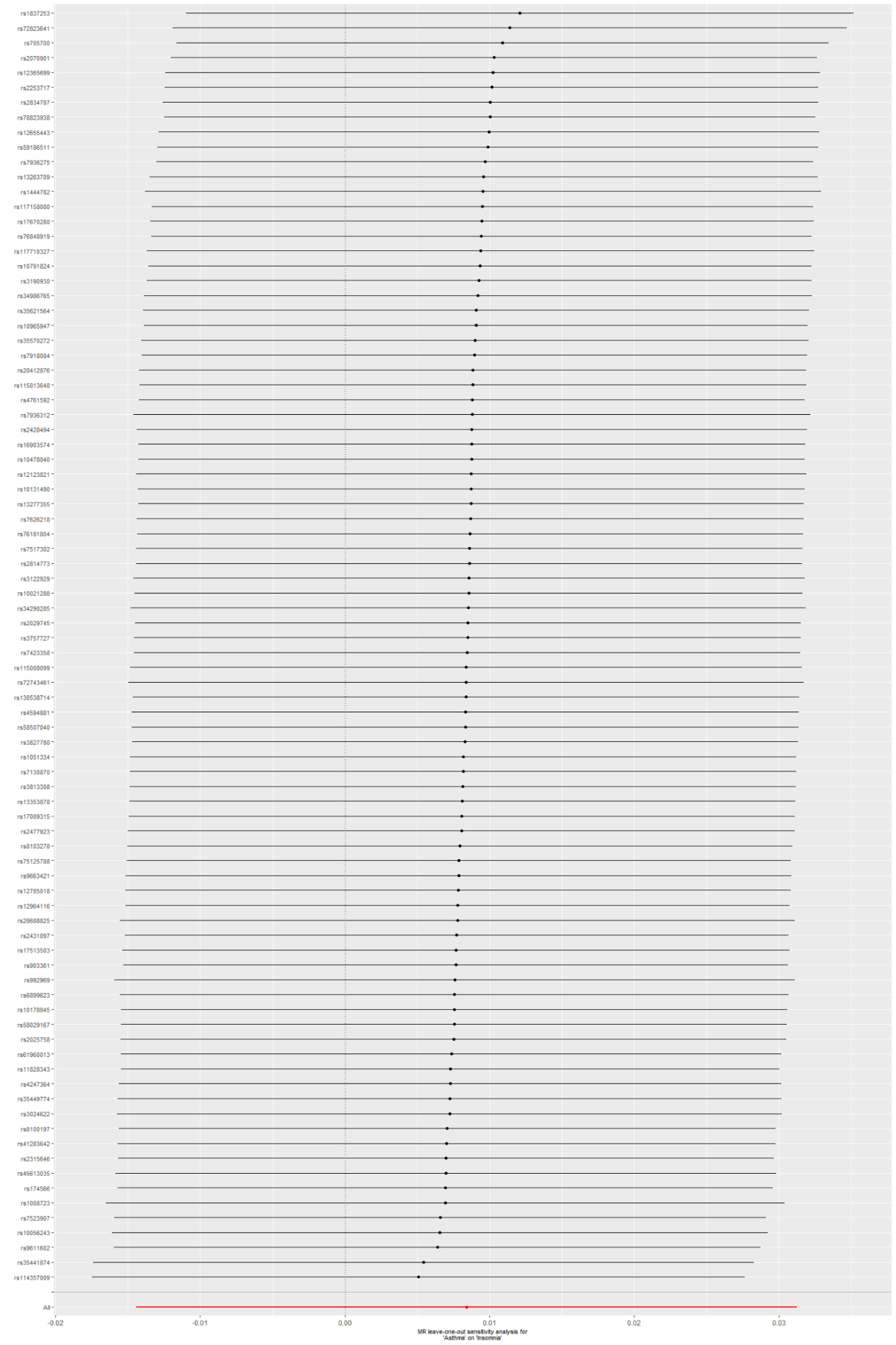
**

**Figure S14** Leave-one-out sensitivity based on IVW model for asthma on insomnia

**
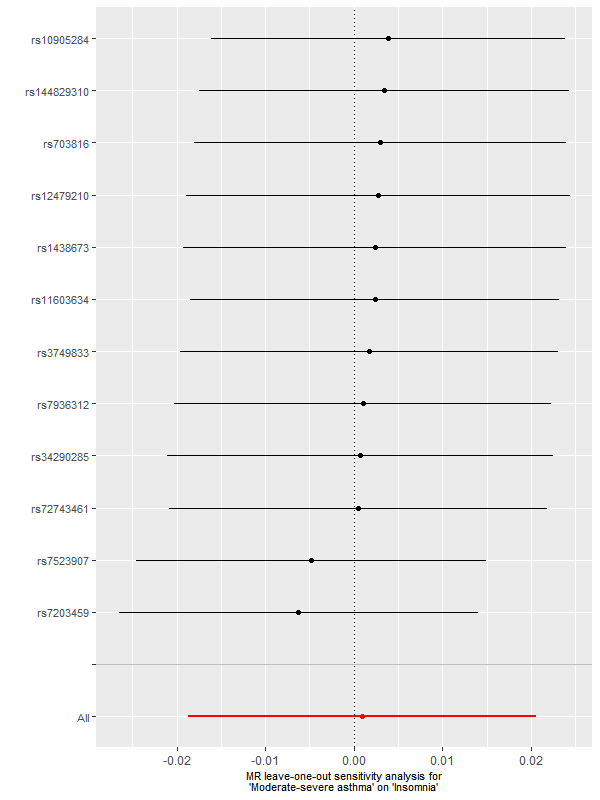
**

**Figure S15** Leave-one-out sensitivity based on IVW model for moderate-severe asthma on insomnia

**
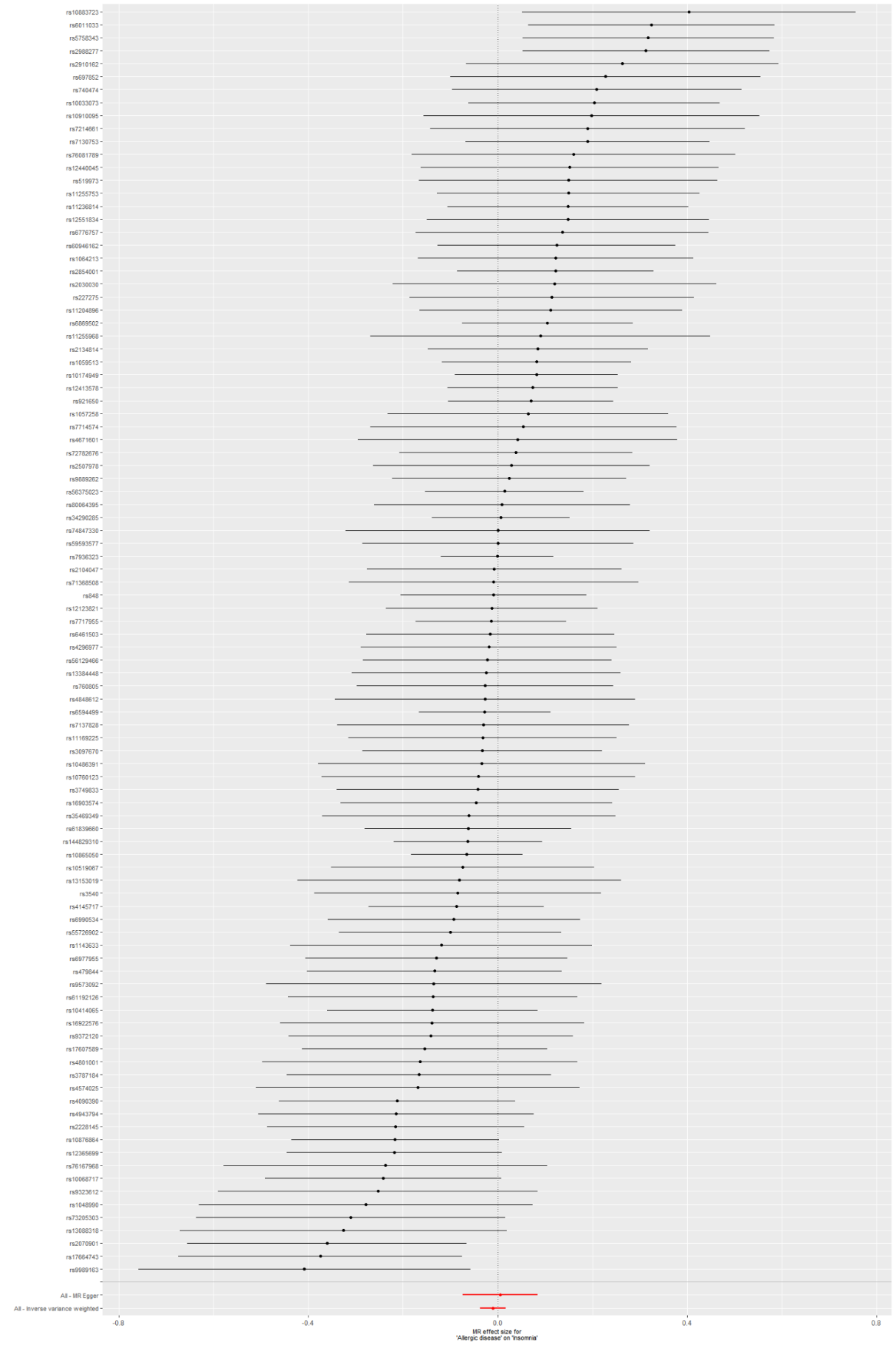
**

**Figure S16** Forest plot for the association between allergic disease and insomnia

**
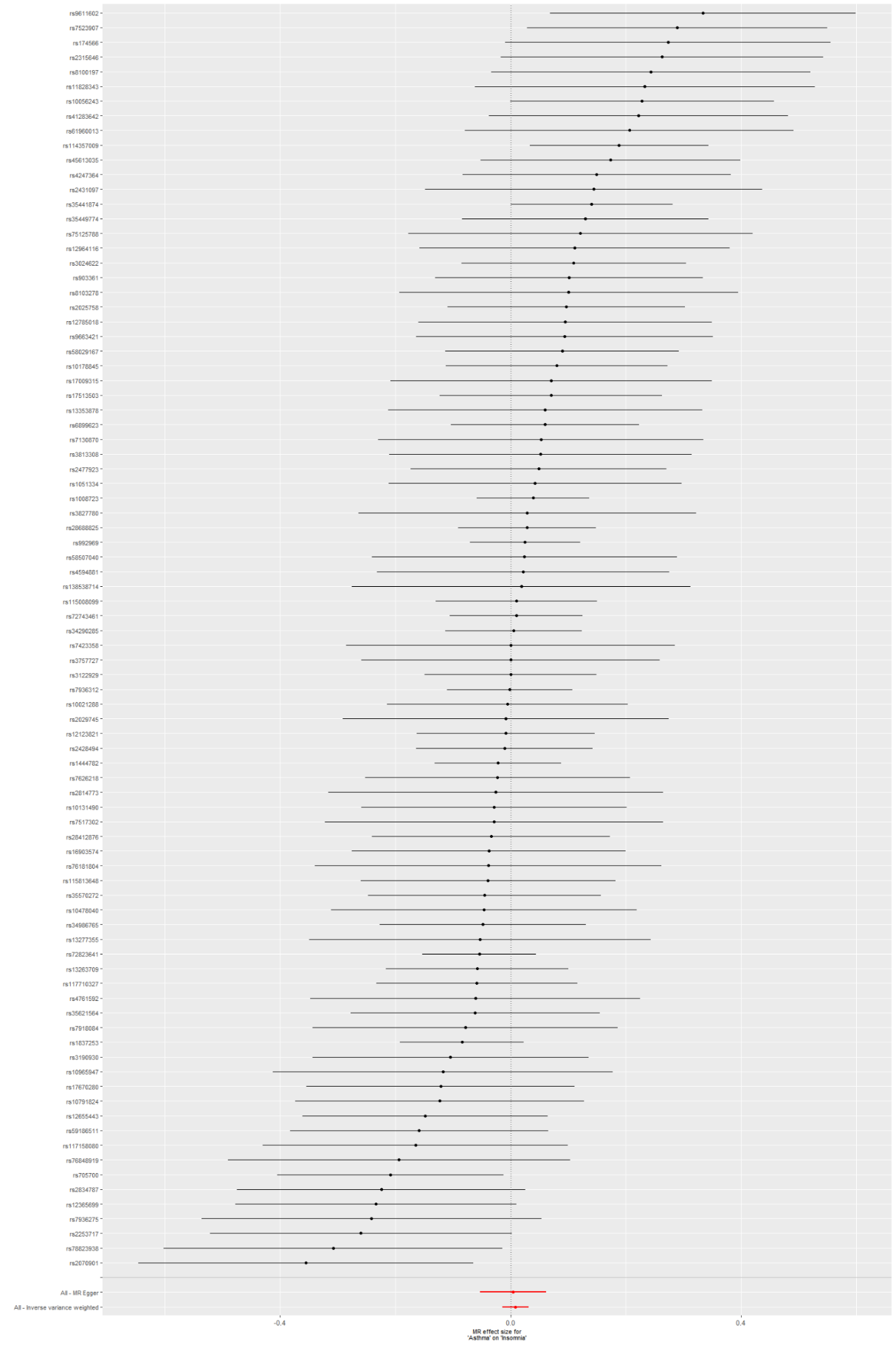
**

**Figure S17** Forest plot for the association between asthma and insomnia

**
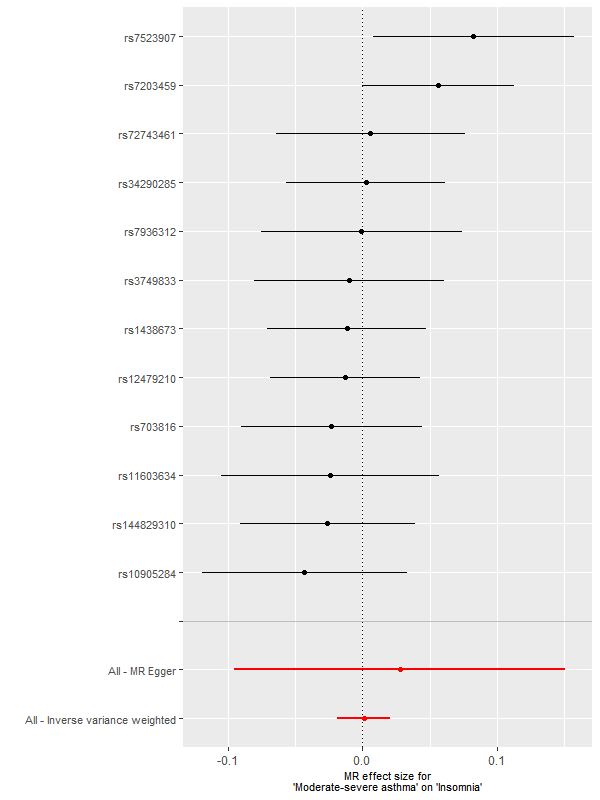
**

**Figure S18** Forest plot for the association between moderate-severe and insomnia
